# The role of the default mode network in longitudinal functional brain reorganization of brain gliomas

**DOI:** 10.1007/s00429-022-02490-1

**Published:** 2022-04-23

**Authors:** Francesca Saviola, Luca Zigiotto, Lisa Novello, Domenico Zacà, Luciano Annicchiarico, Francesco Corsini, Umberto Rozzanigo, Costanza Papagno, Jorge Jovicich, Silvio Sarubbo

**Affiliations:** 1 Center for Mind/Brain Sciences, University of Trento, Corso Bettini, 31-38068 Rovereto, Italy; 2 Department of Emergency, Division of Neurosurgery, Structural and Functional Connectivity Lab Project, “S. Chiara” Hospital, Azienda Provinciale Per I Servizi Sanitari Trento, Trento, Italy; 3 Department of Radiology, Division of Neuroradiology, “S. Chiara” Hospital, Azienda Provinciale Per I Servizi Sanitari Trento, Trento, Italy; 4 Department of Psychology, Milano-Bicocca University, Milano, Italy

**Keywords:** Functional connectivity, Gliomas, Hubs, Default mode network, Cognitive recovery

## Abstract

The study of patients after glioma resection offers a unique opportunity to investigate brain reorganization. It is currently unknown how the whole-brain connectomic profile evolves longitudinally after surgical resection of a glioma and how this may be associated with tumor characteristics and cognitive outcome. In this longitudinal study, we investigate the impact of tumor lateralization and grade on functional connectivity (FC) in highly connected networks, or hubs, and cognitive performance. Twenty-eight patients (17 high-grade, 11 low-grade gliomas) underwent longitudinal pre/post-surgery resting-state fMRI scans and neuropsychological assessments (73 total measures). FC matrices were constructed considering as functional *hubs* the default mode (DMN) and fronto-parietal networks. *No-hubs* included primary sensory functional networks and any other *no-hubs* nodes. Both tumor hemisphere and grade affected brain reorganization post-resection. In right-hemisphere tumor patients, regardless of grade and relative to left-hemisphere gliomas, FC increased longitudinally after the intervention, both in terms of FC within *hubs* (p_hubs_ = 0.0004) and FC between hubs and no-hubs (p_hubs-no-hubs_ = 0.005). Regardless of tumor side, only lower-grade gliomas showed longitudinal FC increases relative to high-grade tumors within a precise *hub* network, the DMN. The neurocognitive profile was longitudinally associated with spatial features of the connectome, mainly within the DMN. We provide evidence that clinical glioma features, such as lateralization and grade, affect post-surgical longitudinal functional reorganization and cognitive recovery. The data suggest a possible role of the DMN in supporting cognition, providing useful information for prognostic prediction and surgical planning.

**Supplementary Information:**

The online version contains supplementary material available at 10.1007/s00429-022-02490-1.

## Introduction

Gliomas are the most common primary tumors of the central nervous system, with an incidence of 4–11 new cases per 100,000 people each year (Bondy et al. 2008). The clinical features can vary, depending on tumor stage and location. The most effective treatment is surgical resection followed by radio and/or chemotherapy (Minniti anzetta 2009; Stupp et al. 2014), with timing dependent on the grading and molecular features. Despite progress in diagnosis and treatment strategies, the overall survival rate is still poor in the case of high-grade gliomas (HGGs) and strongly dependent on timing and extent of resection (EOR) in case of low-grade gliomas (LGGs) (Zigiotto et al. 2020a, b). Oncological prognosis is mostly impacted by the surgical resection in both LGGs and HGGs and is variable based on the molecular characterization (Buckner 2003; Hervey-Jumper and Berger 2014). Consequently, there is an urgent need to formulate more accurate oncological, but also neurocognitive prognoses using non-invasive approaches to provide a basis for individualized treatment and management and to improve the balance between effective resections and preservation of basic and higher cognitive performances (Mandonnet and Duffau 2018).

An important aspect of glioma prognosis is related to how tumor characteristics, such as grade and hemispheric lateralization, may affect cognition and related brain functional networks, particularly given brain reorganization taking place after surgical resection but also during tumor development(Papagno et al. 2011a, b). Such longitudinal brain plasticity processes presumably help sustain cognitive performance and, as such, are crucial for the patient's quality of life.

Two large-scale functional networks are well known as cornerstones in the human connectome (i.e., *hubs*(Crossley et al. 2014; Power et al. 2013; M. P. van den Heuvel and Sporns 2011; Martijn P. van den Heuvel and Sporns, 2013b)) and for sustaining several cognitive processes: the default mode network (DMN) (Raichle 2015), and the fronto-temporo-parietal/attentional network (FTPN) (Zanto and Gazzaley 2013). Relevant cross-sectional works in the field highlighted how DMN aberrantly increases or decreases its functional connections in glioma patients(Esposito et al. 2012; Harris et al. 2014; Tuovinen et al. 2016), whereas FTPN supports cognitive outcome(Lang et al. 2017). Altogether, these findings suggest a crucial role for these *hub* regions in supporting cognitive plasticity (M. P. van den Heuvel and Sporns, 2011), even in glioma patients. However, longitudinal changes of functional *hubs* organization and its relationship with cognitive profiles after brain surgery on gliomas, at the state of the art, have not been extensively studied (see Table S1 for a list of relevant existing studies).

The aim of this longitudinal study was to evaluate with an original and multimodal clinical approach, based on the integration of advanced functional imaging and neuropsychological assessments, how brain glioma variables such as side (right- or left-brain hemisphere) and tumor grade (LGG/HGG) affect brain reorganization and outcome after surgical resection. Specifically, we wanted to assess how these two variables affected the following aspects: (i) functional connectivity (FC) longitudinal changes; (ii) cognitive performance longitudinal changes; and (iii) the relation between functional network longitudinal modifications and cognitive changes.

## Materials and methods

### Participants

Twenty-eight right-handed patients(Oldfield 1971) affected by hemispheric gliomas (22 males; 17 HGG, 11 LGG; age 49 ± 14; educational level 12.2 ± 3.5) who submitted to surgical resection at the Department of Neurosurgery of Santa Chiara Hospital (Azienda Provinciale per i Servizi Sanitari, Trento, Italy) and to adjuvant treatments in case of HGGs (i.e., standard radio- and chemotherapy) participated in this longitudinal study (see Table 1 for all features) during a recruitment period spanning from September 2015 to September 2019, with protocols and techniques previously reported(Zigiotto et al. 2020a, b). Only brain tumor patients with no previous surgery were included in this study. The extent of follow-up was subject to patients’ availability and therefore not standardized across patients, with an overall length reported in Supplementary Table 2.

**Table 1:** Demographic and clinical information of the sample

	Brain tumor types			Statistic	*p*-value
	Low-grade gliomas	High-grade gliomas	Total		
Patients	11	17	28	NA	NA
Age (years mean, SD)	41 ± 15	54 ± 12	49 ± 14*	t(26) = 2.48	0.02*
Gender (# males)	9	13	22	χ2 = 0.11	0.73
Tumor lateralization (# Left)	7	8	15	χ2 = 0.14	0.70
Tumor WHO grade (# patients)	Grade I (2), Grade II (9)	Grade III (5), Grade IV (12)	–	–	–
Tumor volume cm^3 ^(mean, SD)	13.7 ± 3.3	40.3 ± 34.4	26.7 ± 29.5*	t(26) = -2.20	0.037*
Awake surgery (# patients)	10	10	20	χ2 = 1.62	0.20
IDH mutation (# patients)	3	3	6	χ2 = 0.36, NA	0.54, NA
MGMT methylation (# patients)	0	6	6	NA	NA
Extent of resection % (mean, SD)	91.82 ± 11.32	96,68 ± 9.64	94.77 ± 10.41	Z(26) = 1.76	0.08
Radiotherapy [(# patients (weeks mean, SD)]	0	12 (5.75 ± 0.87)	12	NA	NA
Proton therapy [(# patients (weeks mean, SD)]	0	5 (6 ± 0)	5	NA	NA
Chemotherapy [(# patients (months mean, SD)]	0	17 (10.71 ± 3.35)	17	NA	NA
Tumor overlap hubs (mean %, SD %)	1.6 ± 2.2	1.8 ± 2.4	1.4 ± 2.1	t(26) = 1.04	0.30
Tumor overlap no-hubs (mean %, SD %)	2.1 ± 1.9	2.3 ± 1.9	1.7 ± 1.9*	t(26) = 2.23	0.034*

*p-value < 0.05

*SD* Standard deviation, *WHO* World Health Organization, *IDH* Isocitrate dehydrogenase, *MGMT* O6-Methylguanine-DNA-methyltransferase

All neuroradiological and surgical procedures reported are routinely performed according to a protocol approved by the IRBs. All patients gave their informed consent to the surgical procedure and to the use of data for scientific and educational purposes. The study was conducted according to the ethical standards of the Declaration of Helsinki.

### MRI acquisition

Patients were assessed through structural and resting-state functional MRI (rs-fMRI) together with a neuropsychological assessment before, approximately 3-month post-surgery, and then every 3 or 6 months as follow-up (with a maximum of 15 months after surgery, Supplementary Table 2). All images were acquired with a clinical 1.5 T GE Healthcare MRI Scanner at the Department of Radiology of Santa Chiara Hospital, Trento (Italy). For rs-fMRI sessions, patients were instructed to lie still, with eyes open, and to think of nothing. Rs-fMRI images were acquired with 2D T2*-weighted gradient-echo echo-planar imaging (EPI) sequence (TR = 2600 ms, voxel resolution = 4 × 4 × 4.8 mm^3^, TE = 45 ms, FA = 87°, FOV = 256 × 256 mm^2^, # slices = 33–35, acceleration Factor ASSET = 2, TA = 12 min, volumes = 275). Structural scans included standard T1-weighted anatomical (pre- and post-gadolinium) and a T2/FLAIR for every patient (Zacà et al. 2018). A description of MRI pre-processing is reported in Supplemental materials.

### Functional connectome analysis

FC matrices for each patient were constructed at each timepoint of the longitudinal study from the rs-fMRI data using Gordon’s functional parcellation atlas (Gordon et al. 2016), which uses 333 cortical regions in MNI space belonging to different brain functional networks. The MNI atlas was then masked with each subject's GM probability map warped in MNI space. This resulted in a 333 × 333 FC matrix per subject per MRI session, which was created by calculating the absolute value of Pearson correlation coefficients between rs-fMRI time series from all regions. Within each subject, these FC estimates (with values between 0 and 1) were then divided by the subject’s whole-brain averaged FC, to be later used as group-normalized weighted measures of FC between nodes of the network to reduce the potential effect of global FC alterations (Derks et al. 2017; Mueller et al. 2013), which has been seen in glioma patients (Bartolomei et al. 2006; Bosma et al. 2008a, b; Ingeborg Bosma et al. 2008a, b). All FC *hubs* analyses were performed using in-house scripts in Matlab 2019b.

**Fig. 1:**
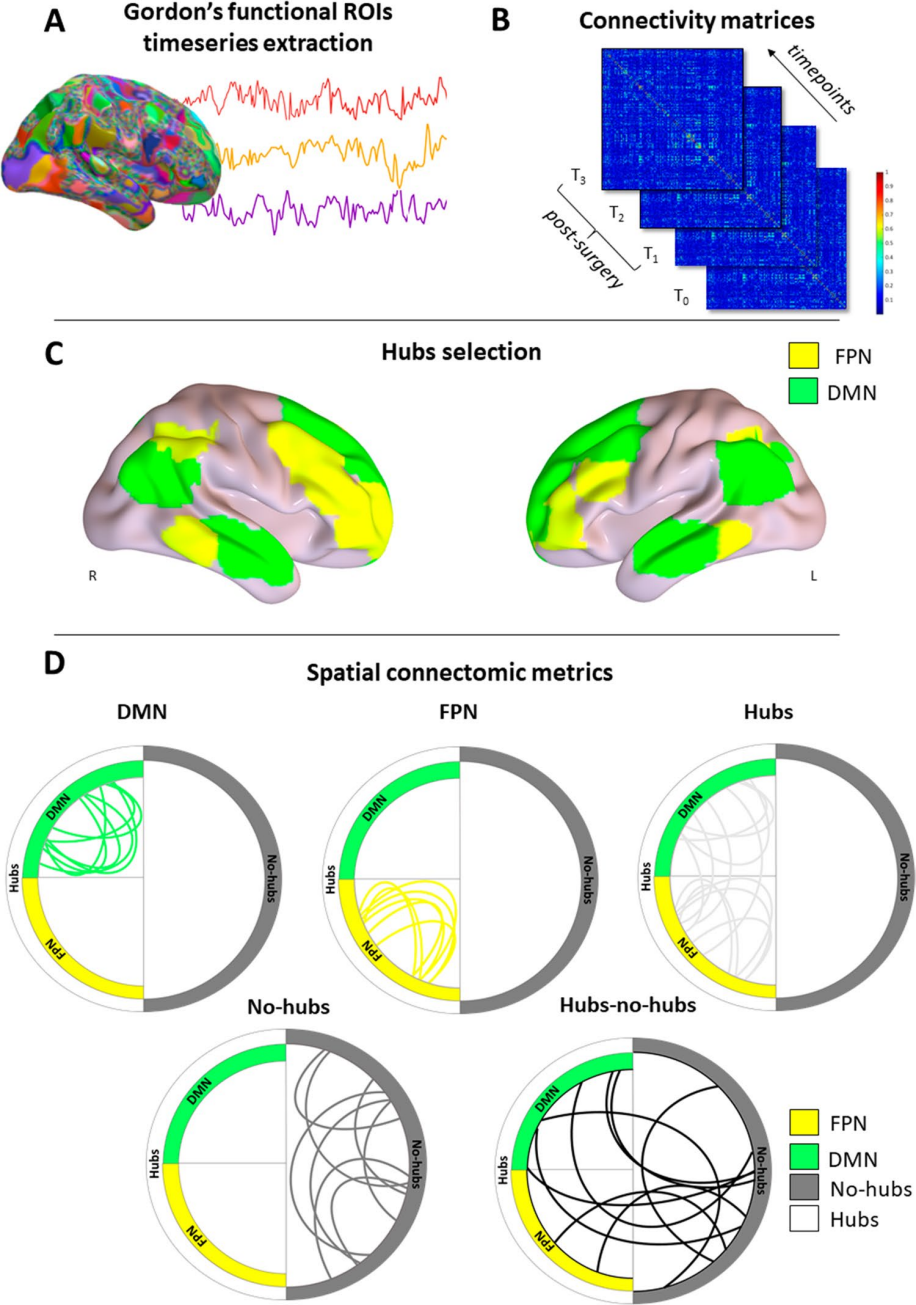
Spatial connectomic pipeline. **A** Functional connectivity (FC) estimation from fMRI time series and Gordon et al. 2016 parcellations. **B** Connectivity matrices computation for each subject at each timepoint. **C** Selection of functional *hubs*: default mode network (DMN), fronto-parietal network (FTPN); **D** Schematic representation, using simulated connections, of the five types of functional connections evaluated: within DMN, within FTPN, within *hubs*, within *no-hubs* and between *hubs* and *no-hubs*

To evaluate the longitudinal post-surgical functional brain reorganization (i.e., the spatial distribution of connectomic profile, Fig. 1), we estimated FC changes in strongly interconnected networks, *hubs* (DMN and FTPN), and in *no-hubs* (primary sensory, such as visual, auditory, and somatosensory, networks as well as salience, cingulate, ventral and dorsal attention). The average FC within and across *hubs* was computed in the standard way, as the average FC value of all possible pairwise voxel correlations from rs-fMRI signal in the predefined regions. Due to the peculiarity of glioma pathology, we defined a priori our *hubs* regions based on previous work in this field (Derks et al. 2017), highlighting how, regardless of the tumor site, DMN and FTPN seem to maintain their functionally central roles in the brain without significant distortions (Figure S1). FC metrics were derived as functional connections: (i) within DMN, (ii) within FTPN, and (iii) within *hubs* (i.e., within FTPN and DMN nodes considered together as a single extended *hub* network), (iv) between *hubs* and *no-hubs,* and (v) within *no-hubs*. Moreover, to estimate the effect of the tumor volume on the parcellation, an overlap index was computed, to measure how many voxels and what percentage of the whole GM atlas consisted of tumor tissue (Table 1 and Supplementary Table 10) with respect to *hubs* or *no-hubs* regions. Finally, FC metrics were then statistically tested with a linear mixed model (see Statistical analysis).

### Neuropsychological assessment

Every patient underwent a longitudinal neuropsychological assessment, including language production, attention, short- and long-term verbal and visual memory, executive function and constructional praxis, as previously reported(Dallabona et al. 2017; Papagno et al. 2011a, b; Zigiotto et al. 2020a, b). The percentage of cognitive deficit, i.e., pathological scores, are reported for every test in Supplementary Tables 4, 5 and 12. Neuropsychological assessment was conducted with a validated battery of tests widely used for LGG and HGG (Dallabona et al. 2017; Papagno et al. 2012; Zigiotto et al. 2020a, b) and performed before surgery (12.96 ± 9.34 days), 1 week after surgery (before hospital discharge), and subsequently every 3 months before or after MRI scanning to be associated with neuroimaging measures.

### Statistical analysis

Statistical analyses were performed using Matlab 2019b and R software (v3.6.0) (https://cran.r-project.org/bin).

Baseline characteristics of the patients were assessed by parametric t-test or non-parametric Mann–Whitney for continuous Gaussian or not-normally distributed variables, respectively (*p* < 0.05), and by Chi-square tests for categorical data (*p* < 0.05). Linear mixed models (*lme4,* R-package, https://www.rdocumentation.org/packages/lme4/versions/1.1-23/topics/lmer) were used to assess the longitudinal dataset. These models tested whether cognitive and FC profiles, regardless of nuisance covariates (i.e., age, tumor volume, IDH mutation, etc..), get differentially affected and how they are longitudinally related in: (i) HGGs patients treated with surgery associated with radio- and chemotherapy and LGGs patients treated with surgery alone over 12 months post-surgery; and (ii) left- and right-lateralized glioma patients over 12 months post-surgery (see Supplementary materials for detailed linear mixed models).

## Results

### Dataset characteristics at baseline

Table 1 depicts the demographic and clinical characteristics of the patient sample. No group difference was found for gender (*χ2* = 0.11, *p* = 0.73), tumor side (*χ2* = 0.14, *p* = 0.70), surgery type (awake/asleep) (*χ2* = 1.62, *p* = 0.20) or extent of resection (*Z*(26) =   –  1.76, *p* = 0.08). Instead, comparing LGGs versus HGGs produced significant group differences for age (LGG < HGG: *t*(26) = -2.48, *p* = 0.02) and tumor volume (cm^3^) (LGG < HGG: *t*(26) =  – 2.20, *p* = 0.037), which were added as nuisance covariates. Since tumor volume was significantly higher in our HGGs sample, we tested for the impact of tumor mass on the a priori* hubs* and *no-hubs* definitions in the parcellation (Gordon et al. 2016). We found that spatial overlap between tumors and either *hubs* or *no-hubs* was small (below 3%, Supplementary Table 10). For this reason, we did not apply tumor masks to the FC analysis (Derks et al. 2017). FC was estimated from gray matter parcellations derived from the T1-weighted images for each subject at each time point (Supplementary materials). Preoperative FC estimations at baseline showed no statistically significant difference between groups (Supplementary materials). Figure S2 depicts tumor sites, respectively, for LGG and HGG with Supplementary Table 11 specifying the anatomical lobe distribution of gliomas in the sample. Concerning neuropsychological assessment, cognitive profile at baseline highlighted no statistically significant differences between groups and is reported divided by tumor grade and by tumor lateralization (Supplementary Tables 4 and 5), and Supplementary Table 6 describes the mean relative longitudinal changes for each subject.

### Hubs functional connectivity

Table 2 reports the results of the linear mixed model analyses showing the variance explained in spatial connectomic profile measures of FC by the fixed effect of *time* (longitudinal differences regardless of the patient's features), *time x tumor grade* interaction (differential longitudinal progression across different grades/treatments) and *time x tumor lateralization* interaction (differential longitudinal progression across different tumor hemispheric lateralization) in the whole population over the observational time (Supplementary Table 2). Spatial connectomic metrics at baseline are described for tumor grade stratification and tumor hemispheric lateralization in Supplementary Tables 7, 8, and Supplementary Table 9 describes the mean relative longitudinal changes for each subject.

**Table 2:** Functional connectivity profile showing significant effects revealed by a linear mixed model of longitudinal changes (baseline, 1–3-, 3–6-, 6–9-, 9–15-month follow-ups) in brain tumor patients stratified into two groups (low-grade gliomas (LGG) correspond to gliomas treated with only surgical resection, while high-grade gliomas (HGG) correspond to gliomas treated with surgical resection in combination with radiotherapy and/or chemotherapy). The model included the following main predictors: *time* (a positive effect means longitudinal connectivity increases regardless of tumor grade and lateralization), *time* ×*tumor grade* interaction (a positive effect means that longitudinal connectivity increases faster in LGG relative to HGG, regardless of lateralization) and *time* ×*tumor lateralization* (positive means that longitudinal connectivity in left-lateralized glioma increases faster than in right-lateralized glioma, regardless of tumor grade). The model was adjusted with age, sex, baseline tumor volume, IDH mutation, tumor methylation, and tumor WHO grade as nuisance variables. Significant (*p < *0.05) fixed effects are emphasized in bold with their respective effect sizes and an asterisk if the effect survives FDR correction across different models

Response	Predictors [Estimate β, *p*-value, Cohen’s d if significant]			Random effects
	Time	Time × tumor grade	Time × tumor lateralization	σ^2^
Within default -mode network	[0.003, 0.54]	[0.00002, 0.01, 0.001]*	[0.006, 0.001, 0.28]*	0.01
Within fronto-parietal network	[0.006, 0.28]	[ – 0.01, 0.63]	[ – 0.003, 0.11]	0.01
Within hubs	[0.002, 0.68]	[ – 0.003, – 0.15]	[0.005, 0.0004, 0.29]*	0.01
Within no-hubs	[ – 0.0006, 0.44]	[0.0010, 0.71]	[ – 0.001, 0.004, – 0.30]*	0.0004
Between hubs-no-hubs	[0.0004, 0.75]	[ – 0.001, 0.65]	[0.0023, 0.005, 0.40]*	0.0001

*p-value_FDR_ < 0.05

**Fig. 2:**
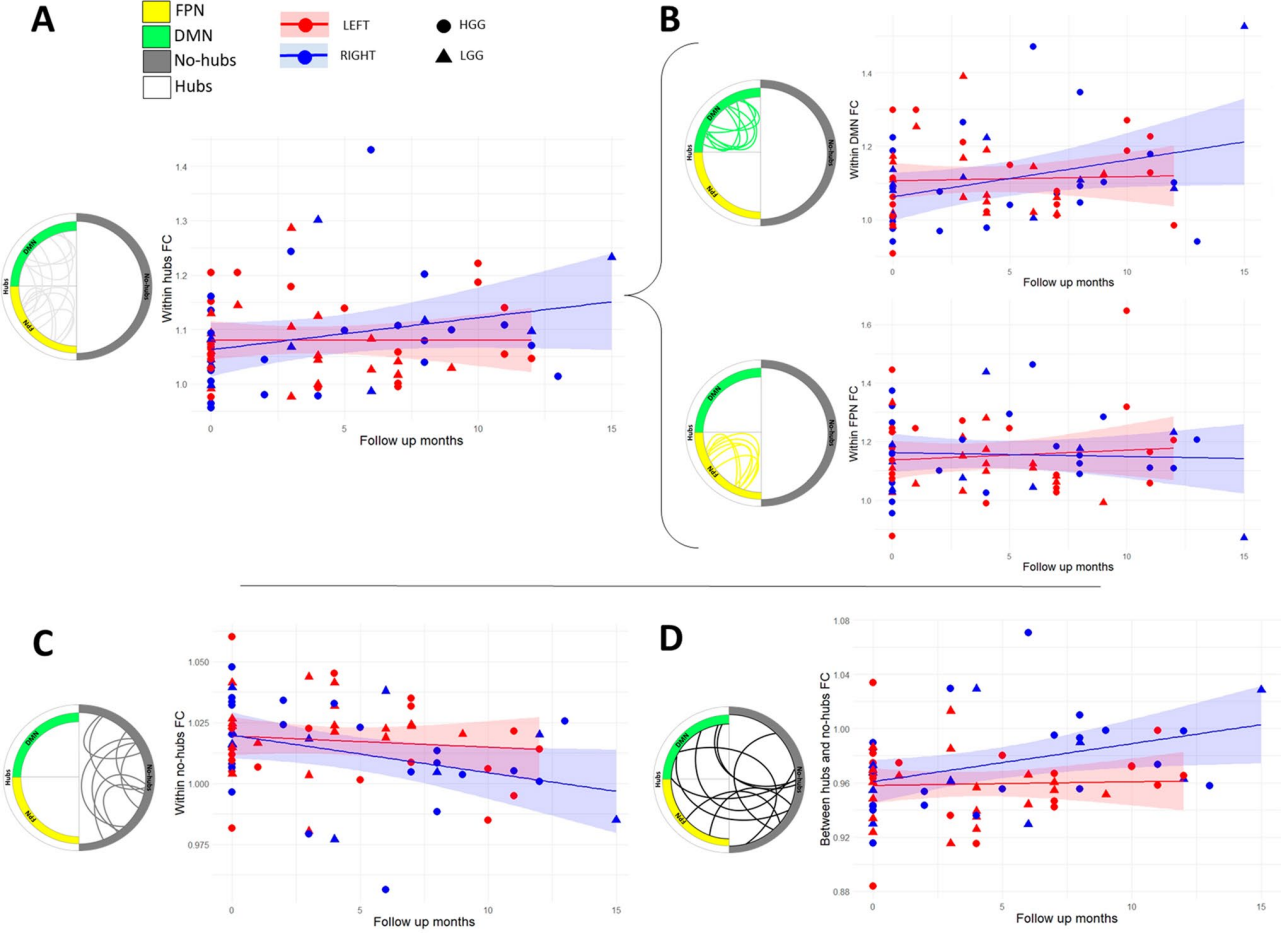
Significant longitudinal functional connectivity (FC) profile changes per subgroup. The longitudinal trend for all connectivity profiles of brain tumor patients, specified according to tumor lateralization (i.e., left and right hemispheres). **A** Average normalized connectivity within *hubs* over time. **B** Average normalized connectivity within default mode network (DMN) and within fronto-parietal network (FTPN; not significant) over time. **C** Average normalized connectivity within *no-hubs* and **D** between *hubs* and *no-hubs* over time. Circular plots represent, using simulated connections, the five types of functional connections evaluated: within DMN, within FTPN, within *hubs*, within *no-hubs* and between *hubs* and *no-hubs*

As far as *time* is concerned, regardless of glioma patients’ characteristics, the fixed effect did not show any significant effect (*p* > 0.05). *Time x tumor grade* interaction effect was significant only when considering within-DMN FC (*p* = 0.01, *Std β* = 0.00002), with LGGs associated with larger within-DMN FC over time. Figure 2 shows the significant *time x tumor lateralization* interaction effect for the four spatial connectomic profile measures (i.e., within *hubs*, within *no-hubs*, within DMN, and between *hubs* and *no-hubs*). The four subplots display the mean predicted values of above-cited FC measures in the two different subgroups of glioma patients, per tumor hemispheric location over the 15 maximum months of assessment. Overall, in right-lateralized tumor patients the FC profile increases longitudinally within *hubs* (*p* = 0.0004, Std β = 0.005) relative to FC in left-lateralized tumor patients (Fig. 2A), and this longitudinal gain in FC seems to be mainly driven by within-DMN FC over time (*p* = 0.001, *Std β* = 0.006; Fig. 2B), since no significant effect was found for within-FTPN FC. Moreover, the longitudinal evolution of FC within left- and right-lateralized FTPN were tested separately, resulting in no significant effect relative to the whole FTPN analysis. However, *no-hubs* regions are characterized by an opposite trend, with a steeper longitudinal decay for right-lateralized tumors (*p* = 0.004, *Std β* =  – 0.001; Fig. 2C), as compared to those left-lateralized. Lastly, regarding connectivity changes between different networks, FC between *hubs* and *no-hubs* largely increases over time in right-lateralized tumors (*p* = 0.005, *Std β* = 0.002; Fig. 2D).

### Neurocognitive profile

Mean scores for each test at the different time points and percentages of deficit are reported in Supplementary Table 12. Table 3 reports the results of the linear mixed model showing the variance explained in cognitive profile measures by the fixed effect of *time* (longitudinal differences regardless of the patient's features), *time × tumor grade* interaction (differential longitudinal progression across different tumor grades/treatments), and *time x tumor lateralization* interaction (differential longitudinal progression across different tumor side) in our sample over the observational time (Supplementary Table 2).

**Table 3:** Neuropsychological profile showing significant effects revealed by a linear mixed model of longitudinal changes (baseline, 1–3-, 3–6-, 6–9-, 9–15-month follow-ups) in brain tumor patients stratified into two groups (low-grade gliomas (LGG) correspond to gliomas treated with only surgical resection and high-grade gliomas (HGG) correspond to gliomas treated with surgical resection in combination with radiotherapy and/or chemotherapy). The model included the following main predictors: *time* (a positive effect means that, longitudinally, the neuropsychological measure increases regardless of tumor grade and lateralization), *time* × *tumor grade* interaction (a positive effect means that, longitudinally, the neuropsychological measure increases faster in LGG relative to HGG, regardless of lateralization) and *time* × *tumor lateralization* (positive means that, longitudinally, the neuropsychological measure in left-lateralized glioma increases faster than in right-lateralized glioma, regardless of tumor grade). The model was adjusted by age, sex, baseline tumor volume, IDH mutation, tumor methylation, and tumor WHO grade as nuisance variables. Significant (*p* < 0.05) fixed effects are emphasized in bold with their respective effect size and an asterisk if the effect survives FDR correction across different models of the same cognitive domain

Response	Predictors [Estimate β, *P*-value, Cohen’s d (if significant)]			Random effects
	Time	Time × tumor grade	Time × tumor lateralization	σ^2^
Rey complex figure delayed reproduction	[0.75, 0.0025, 0.94]*	[ – 0.26, 0.51]	[ – 0.26, 0.45]	25.81
Rey’s 15-word list delayed recall	[ – 0.07, 0.432]	[ – 0.14, 0.12]	[0.18, 0.032, 0.43]	3.95
Picture-naming test	[0.14, 0.15]	[ – 0.09, 0.0333, – 0.22]	[0.08, 0.74]	3.94
Corsi span	[ – 0.04, 0.15]	[0.07, 0.0435, 0.57]	[0.06, 0.638]	0.34
Semantic fluency	[ – 0.55, 0.046, – 0.63]	[0.42, 0.66]	[0.82, 0.17]	31.93

*p-value_FDR_ < 0.05

**Fig. 3:**
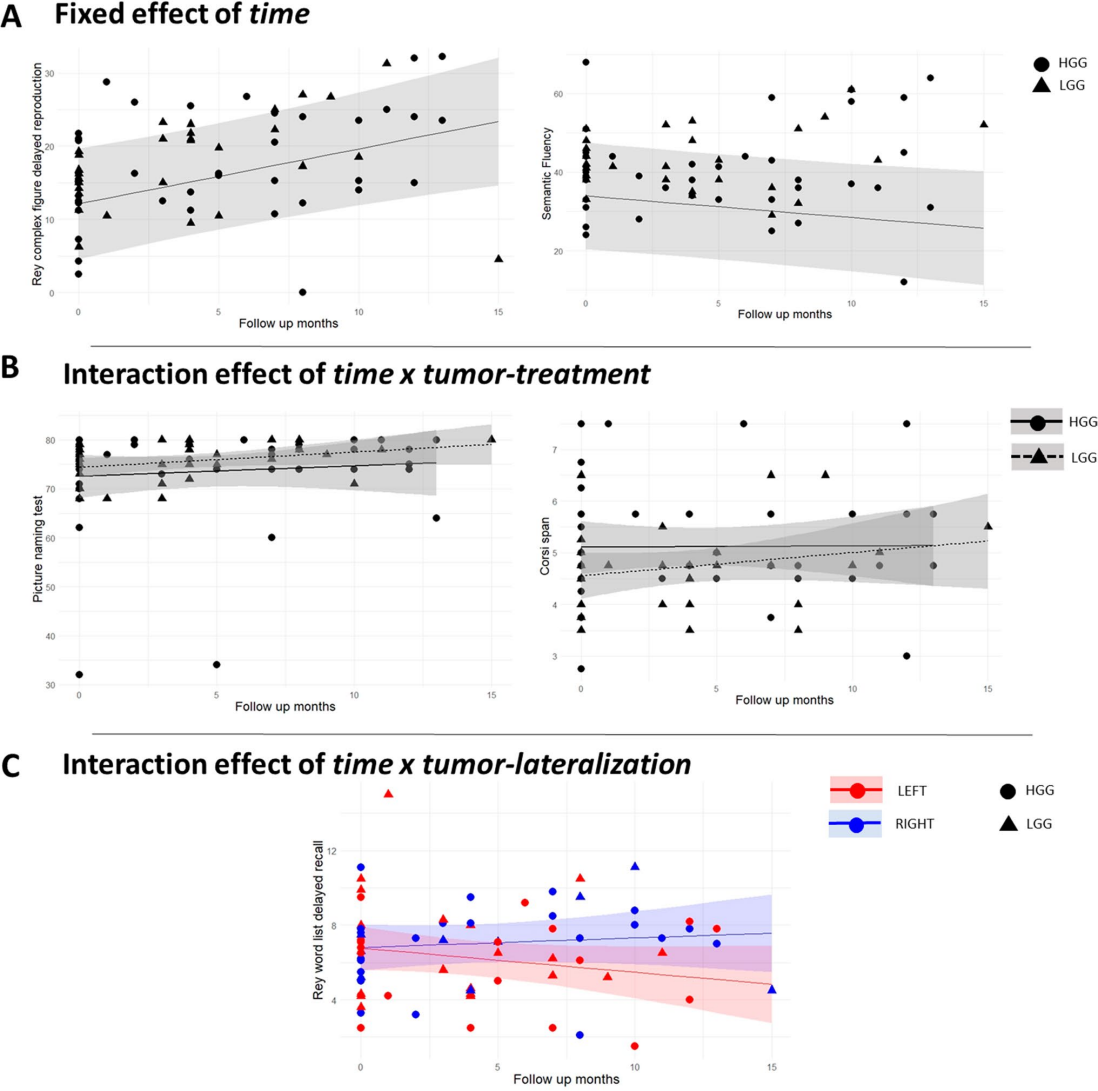
Significant longitudinal neuropsychological profile changes. The longitudinal trend for the neuropsychological profile of brain tumor patients according to grade (low-grade gliomas (LGG); high-grade gliomas (HGG)) and tumor lateralization (i.e., left and right hemispheres). **A** fixed effect of *time* for Rey complex figure delayed reproduction and Semantic fluency. **B** fixed interaction effect of *time* with *tumor grade* for Picture-naming test and Corsi span, respectively. **C** fixed interaction effect of *time* with *tumor laterality* for Rey’s 15-word list delayed recall

As far as *time* is concerned, regardless of tumor side and grade, the fixed effect showed positive significant effects in long-term visual memory (Fig. 3A; Rey complex figure delayed reproduction: *p* = 0.0025, *Std β* = 0.75), meaning a better performance over time; however, considering the use of the same complex figure, this result may reflect a learning effect across sessions. More relevantly, we observed a negative effect in verbal fluency (Fig. 3A; Semantic fluency: *p* = 0.046 uncorrected, *Std β* =  – 0.55), which worsened over time. The *time x tumor grade* interaction effect was significant, with LGGs showing a better performance for short-term visuospatial memory (Fig. 3B; Corsi span: *p* = 0.043 uncorrected, *Std β* = 0.07) and a reversed pattern for naming (Fig. 3B; Picture-naming test: *p* = 0.0033 uncorrected, *Std β* =  – 0.09). Finally, a significant *time x tumor lateralization* interaction effect was found, with right-lateralized tumors characterized by an improvement, as compared to those left-lateralized, selectively for long-term verbal memory, as one might expect (Fig. 3C; Rey’s 15-word list delayed recall: *p* = 0.032 uncorrected, *Std β* = 0.18).

### The neurocognitive outcome predicted by hubs functional connectivity

Linear mixed models were then used to test for associations between connectomic spatial profiles and cognitive scores. These models were used to assess how a longitudinal change in FC could impact the prediction of the cognitive score, associating the observed connectomic spatial information over time and the corresponding behavioral outcome.

**Fig. 4:**
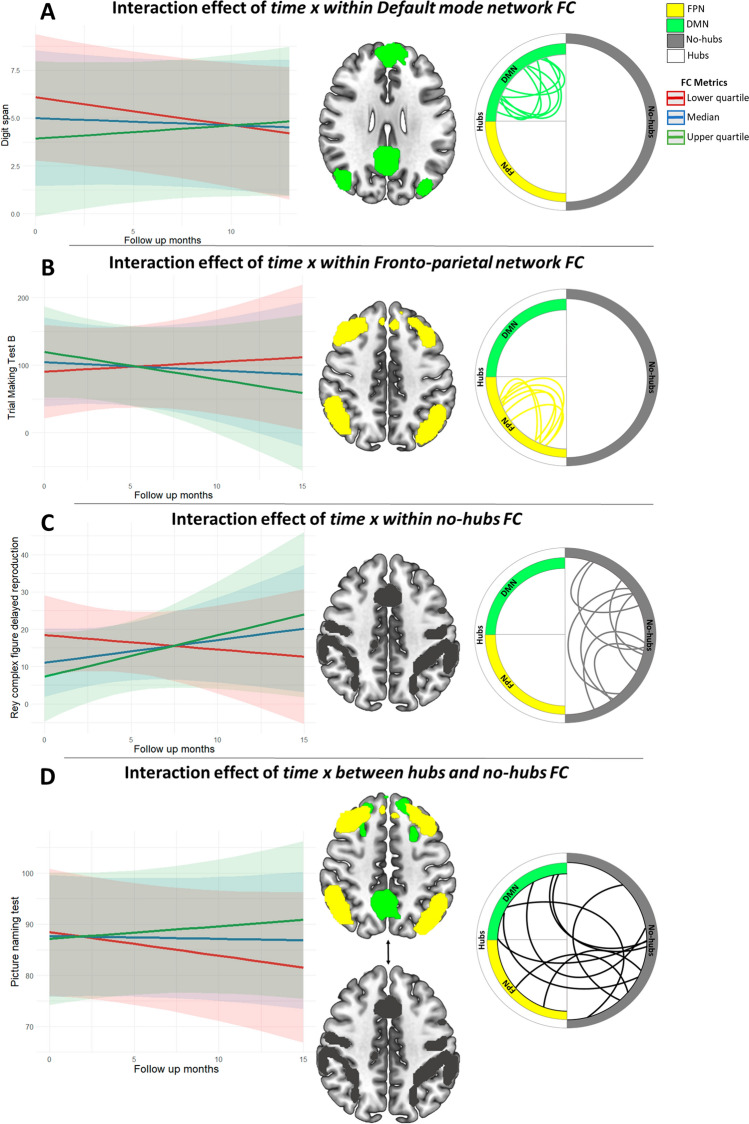
Prediction of the neuropsychological profile from longitudinal changes in functional connectivity metrics. Interactions effect for the neuropsychological profile of brain tumor patients, regardless of WHO tumor grade and tumor lateralization, by plotting the quartiles of the distribution of each FC metric. **A** Fixed interaction effect of *time* with default mode network (DMN) for Digit span. **B** Fixed interaction effect of *time* with fronto-parietal network (FTPN) for Trial Making Test B. **C** Fixed interaction effect of time with *no-hubs* for Rey complex Figure delayed reproduction and **D** between *hubs* and *no-hubs* for Picture naming. Circular plots represent, using simulated connections, the five types of functional connections evaluated: within DMN, within FTPN, within *hubs*, within *no-hubs* and between *hubs* and *no-hubs*

Interestingly, the interactions between several FC measures and time were significantly associated with several cognitive scores (Table 4 and Fig. 4). A significant interaction effect between *time x FC within DMN* was found for short-term verbal memory score (Fig. 4A; Digit span: *β*_DMN*time_ = 0.32, *p*_DMN*time_ = 0.008), where higher FC within DMN over time predicts patients’ higher cognitive performances. Similarly, the trend of FC within *hubs* over time also seems relevant for supporting attentional abilities: for Trial Making Test A and B (TMT-A and -B), a significant interaction effect was found between *time x within both DMN FC* (Figure S3A; TMT-A: *β*_DMN*time_ =  – 12.75, *p*_DMN*time_ = 0.016; TMT-B: *β*_DMN*time_ =  – 25.90, *p*_DMN*time_ = 0.019) and *FTPN FC* (Figure S3B and Fig. 4B; TMT-A: *β*_FTPN*time_ =  – 2.80, *p*_FTPN*time_ = 0.0007; TMT-B: *β*_FTPN*time_ =  – 20.20, *p*_DMN*time_ = 0.042). This highlighted how patients with both higher FC within DMN and within FTPN over time are characterized by a better cognitive performance in divided attention (TMT-A and TMT-B). For long-term visual memory, we found a significant interaction effect between *time x no-hubs FC* (Fig. 4C; Rey complex figure delayed reproduction: *β*_no-hubs*time_ = 49.7, *p*_hubs-no-hubs*time_ = 0.035 uncorrected). On the other hand, naming improvement seemed to be associated with patients characterized by an increase in FC between *hubs* and *no-hubs* regions over time (Fig. 4D; Picture-naming test: *β*_hubs-no-hubs*time_ = 10.1, *p*_hubs-no-hubs*time_ = 0.0083). All the above-reported associations, besides the prediction of long-term visual memory based on the FC profile of *no-hubs* over time (see Limitations), are consistent with the observed FC changes over time, making these neural longitudinal changes classifiable as potentially resilient.

**Table 4:** Neuropsychological profile showing significant effects revealed by a linear mixed model of longitudinal changes (baseline, 1–3-, 3–6-, 6–9-, 9–15-month follow-ups) associated with Functional Connectivity (FC) in brain tumor patients stratified into two groups (low-grade gliomas (LGG) correspond to gliomas treated only with surgical resection and high-grade gliomas (HGG) correspond to gliomas treated with surgical resection in combination with radiotherapy and/or chemotherapy). The model included time and *time* × *FC metrics* (within default mode network (DMN) FC, within fronto-parietal network (FTPN) FC, within *no-hubs* FC, between *hubs* and *no-hubs* FC) interactions as main predictors of interest (a positive effect means longitudinal neuropsychological measure increases with the spatial connectivity measure regardless of tumor grade and lateralization) adjusted by age, sex, baseline tumor volume, IDH mutation, tumor methylation and tumor WHO grade as nuisance variables. Significant (*p* < 0.05) fixed effects are emphasized in bold with their respective effect sizes and an asterisk if the effect survives FDR correction across different models of the same cognitive domain

Response	Predictors [Estimate β, *P*-value, Cohen’s d (if significant)]				Random effects
	Time × within DMN FC	Time × within FTPN FC	Time × within no-hubs FC	TIME × between hubs-no-hubs FC	σ^2^
Rey complex figure delayed reproduction	[ – 0.85, 0.08]	[1.28, 0.66]	[49.7, 0.035, 0.36]	[20.16, 0.34]	21.33
Digit span	[0.32, 0.008, 0.36]*	[0.38, 0.25]	[12.9, 0.099]	[5.71, 0.07]	0.38
Picture-naming test	[ – 0.88, 0.77]	[0.30, 0.35]	[2.42, 0.23]	[10.1, 0.0083, 0.39]*	4.13
Trial-making test A	[ – 12.75, 0.016, – 1.16]*	[ – 2.80, 0.0007, – 0.30]*	[ – 163.35, 0.28]	[ – 14.51, 0.12]	61.77
Trial-making test B	[ – 25.90, 0.019, – 0.63]*	[ – 20.20, 0.0425, – 0.57]	[ – 504.97, 0.09]	[ – 157.10, 0.12]	831.10

*p-value_FDR_ < 0.05

## Discussion

Using functional neuroimaging and neuropsychological evaluations, this study reveals longitudinal brain functional reorganization during the follow-up of two groups of glioma patients undergoing standard treatment protocols. By investigating FC changes focusing on *hubs* regions, we provide new evidence about longitudinal functional plastic changes over time and confirm previous results in the field (Derks et al. 2017; Tuovinen et al. 2016). We show: (i) at a neural level, brain network *hubs* exhibit the strongest FC changes, with a mild effect of tumor grade (HGG show less longitudinal connectivity increase than LGG) but a large effect of tumor lateralization (left tumors show a lower level of reorganization), and ((ii) at a cognitive level, neuropsychological performance shows several effects of time in memory, language, and attentional domains, depending on tumor lateralization and grade.

Here, we show how brain functional plastic reorganization takes place, presumably to sustain cognitive performance in the long term. Two tumor features emerged as factors that differently affect the functional reorganization process. First, tumor grade (together with aggressive post-surgical treatment) has a mild impact on *hubs* functional reorganization over time, which goes partially beyond the distinction based exclusively on the temporal evolution of such lesions (Desmurget et al. 2007). Second, left lateralization was demonstrated to be a strong negative outcome predictor of this plastic functional reshaping and, thus, should be considered in treatment planning. This is in line with the worse cognitive performance of patients with left gliomas, compared to those with right ones (Habets et al. 2019).

Therefore, it is clear from the present results that longitudinal functional reorganization of *hubs* regions provides new insights into the mechanisms underlying progression of different glioma types, into outcomes after surgical resection, and into the impact of functional plasticity changes in sustaining cognitive recovery.

### Glioma grade effects on post-surgery longitudinal functional brain reorganization

The potential of functional reorganization in gliomas has been historically and cross-sectionally associated with the growing nature of the lesion (Desmurget et al. 2007) and its biological aggressiveness, where lesser extension of the mass was associated with slower plastic functional changes (i.e., LGGs compared to HGGs), resulting in more resilient connections (Esposito et al. 2012). In this study, we show a similar longitudinal reorganization pattern at the whole-brain level in different tumor grades and treatments. However, we show that within-DMN FC, HGGs’ reorganization is weaker relative to that of LGGs. Overall, this highlights how, regardless of the tumor's histopathological characteristic, functional plasticity takes place after surgery in the whole connectome.

Previous findings have cross-sectionally highlighted how HGGs show reduced adaptive functional reorganization, which results in greater cognitive impairments relative to LGGs, both before and after surgery (Dallabona et al. 2017; Raysi Dehcordi et al. 2013). By longitudinally looking at postoperative cognitive performance, we observed that, at about one year after surgery, the cognitive outcome is similar regardless of tumor grade in verbal memory, executive functions, attention, visuospatial and constructional praxis skills. The key difference that distinguishes HGGs from LGGs, in association with various tumor treatments, is within-DMN FC, which shows a lower longitudinal increase in HGG relative to LGG. Cross-sectional studies in glioma patients describe DMN to be mainly disrupted, as compared to healthy controls Esposito et al. 2012), with a negative impact of HGGs on its FC (Harris et al. 2014). In our cohort, we demonstrate that LGGs show a postoperative longitudinal connectivity increase within DMN. This effect was absent in HGG: interestingly, besides its function in episodic verbal memory retrieval and memory consolidation (Sestieri et al. 2011), this observed alteration may support DMN’s role in short-term verbal memory, as demonstrated in our cohort, where higher FC within DMN over time predicts higher patient performance.

From a clinical perspective, these results highlight the importance of intra-hyper-connected networks (e.g., *hubs*) for sustaining cognitive recovery in glioma patients after surgery. We found this to be particularly important for DMN, since FC within FTPN was not affected by tumor grade nor by cognitive changes. However, considering that HGGs patients undergo additional adjuvant treatments, we cannot disentangle tumor grade from treatment effects. Indeed, radiotherapy is thought to impact not only specific FC patterns in terms of larger reduction of DMN (Harris et al. 2014) with intact whole-brain networking (Tuovinen et al. 2016), but also in terms of worse cognitive performance (Klein et al. 2012).

### Glioma lateralization effects on post-surgery longitudinal functional brain reorganization

Another relevant property to be considered is the hemispheric localization of the tumor, which has been extensively studied (Stoecklein et al. 2020). Indeed, left-lateralization is thought to be a predictor of both reduced brain connectivity changes (Maesawa et al. 2015; Voets et al. 2019) and impaired cognition (Satoer et al. 2013).

Our study confirmed the relevance of tumor hemispheric lateralization. A right-hemisphere glioma, regardless of its grade and treatment, seems to longitudinally increase FC in *hub* regions compared to left-lateralized ones. Indeed, in the case of glioma in the right hemisphere, *hubs* (especially DMN nodes) reinforce FC within their networks and increase FC to *no-hubs* regions, potentially as a response to improving the integration of whole-brain networking. At the same time, these patients showed a decrease in FC within *no-hubs* regions. In the presence of a glioma, we can suggest that the brain undergoes maladaptive mechanisms due to the disease spreading, which will then impact its FC. But after surgical resection of the tumor, right-lateralized gliomas show a high predisposition to recreating the original physiological situation, in which: (i) high central nodes (i.e., *hubs*; heteromodal cortices) have a higher number of connections, (ii) nodes with lower centrality have lesser connections (i.e., *no-hubs*; sensory cortices), (iii) and connections between them are increased in favor of network integration (i.e., between *hubs* and *no-hubs* FC). Our study shows that left-lateralized gliomas disrupt brain FC ability to plastically return to a system promoting integration between highly segregated and central areas(Martijn P. van den Heuvel and Sporns 2013a), which was previously described only cross-sectionally (Maesawa et al. 2015). From a cognitive perspective, we observed an improvement in verbal long-term memory in patients with right-lateralized tumors: this result supports the hypothesis of the role of DMN in memory consolidation and reinforces our previously discussed results regarding the better-observed *hubs* FC for right- compared to left-lateralized tumors. However, it must be noted that verbal memory assessment involves language abilities and speech production (Bogaerts et al. 2015), which may be affected by a left-hemisphere tumor resection.

From a clinical point of view, the reduced functional reorganization ability of left-lateralized glioma should be considered while planning the treatment by stressing the importance of maintaining not only primary network FC (i.e., *no-hubs*) functionally intact during surgery but also their higher-function counterpart (i.e., *hubs*), which was demonstrated to be relevant for sustaining cognition. In fact, our results show how left-hemisphere glioma pervasively disturbs whole-brain FC impact both within-networks and between-network connections.

### Relevance of key functional networks for neurocognitive recovery

A growing consensus in the neuroscientific community has considered brain *hubs* as regions subserving multiple cognitive abilities (Weaver 2015). In this framework, our results provide new evidence not only for the role of hyper-connected regions (i.e., *hubs*) to plastically re-establish physiological functional networking in gliomas but also for their associations with the post-surgical recovery of cognitive functions, especially attention, naming, and memory abilities.

As previously cited, one of the most connected functional networks or *hubs* of the brain is DMN, which has been shown to be related to different cognitive processes, from mind-wandering to memory (Fox et al. 2005). On the other side, the hyper-connected system of FTPN, is considered a more *“executive” hub* involved in attentional processing (Marek and Dosenbach, 2018). However, large-scale functional networks may communicate among each other, through between-network connectivity, to support a required cognitive outcome. Indeed, in this picture, DMN is thought to play a cardinal role (Gordon et al. 2020), with its different sub-part networks sustaining distinct connections to specific networks for a defined cognitive behavior, conjointly with the FTPN. By looking at FC within *hubs* (DMN and FTPN jointly), we found increased cognitive performance in the attentional domain associated with an increase of FC within *hubs* over time. Without data supporting the causality of such associations, we may only speculate about their possible nature. One possibility is that, given the FTPN role as a flexible cognitive control network (Marek and Dosenbach, 2018), its FC with DMN may need reinforcement to improve attention and thus recover control of the FTPN (De Baene et al. 2020; Douw et al. 2016). This idea is further supported by the known presence of connection streams between the FTPN and sub-parts of the DMN (Gordon et al. 2020) and their causal role in cognitive flexibility (Mandonnet et al. 2020). In addition, the DMN is believed to interact with the language network (van Dokkum et al. 2019). Indeed, we found language abilities, such as naming, to be positively affected in glioma patients by an increase of FC between *hubs* and *no-hubs* over time. These findings suggest that linguistic skills may be supported by the increased FC between hub regions (mainly DMN) and language-specific regions. Such network interactions have been hypothesized to represent internal linguistic inputs Bzdok et al. 2016; Gordon et al. 2020). With regard to memory outcome, verbal short-term memory scores (Baddeley 1992) are positively associated with FC within DMN over time. Considering this result and the role of the DMN in working memory tasks (Santangelo and Bordier 2019), preserving this network may lead to better cognitive recovery after surgery (Kocher et al. 2020).

Overall, the clinical relevance of these findings relies on the association between the longitudinal trend of *hubs* FC and neuropsychological outcome. Indeed, we can interpret as resilient and efficient most of the functional network changes observed: (i) the longitudinal increase in within-*hubs* FC found in right-lateralized and low-grade tumors can potentially be associated with an increased attentional and short-term memory performance; and (ii) the longitudinal increase in between-*hubs*-and-*no-hubs* FC found in right-lateralized tumors can potentially be associated with better naming performance.

Our study suggests that including functional mapping of *hubs* as part of clinical treatment planning may improve patient cognitive outcomes by preserving relevant functional connections, especially in the case of left-lateralized tumors. In particular, pre-surgical mapping of DMN seems important when planning HGG interventions, since these are tumors with unfavorable prognoses and with less time for recovery. For this reason, whenever possible, preserving DMN and its modulation of the FTPN in such patients may lead to better preservation of neurocognitive performance after the intervention. This will improve the quality of life over the history of the disease. Further studies are needed to better understand how FC in *no-hubs* is affected by glioma and its relationship to postoperative cognitive outcomes.

### Limitations

This study had several limitations. First, the sample size. Despite the strength of having an unprecedented longitudinal design (total of 73 observations), the need for follow-up sessions limited our patient sample (*N = *28). Moreover, the follow-up period ended at a maximum of 15 months, not allowing for the investigation of neural plastic changes at longer intervals (2–3 years) post-surgery. Second, tumor anatomical sites and volumes were variable. This concern was reduced given the minimal extent of tumor overlap with functional parcellations (< 2%). However, given the sparse anatomical distribution of the left/right-lateralized tumors in relation to *hubs,* we cannot exclude the influence of low sample-size effects on these results. Moreover, clinical features were controlled and added as covariates in the statistical models. Third, the number of neuropsychological tests was limited, especially for language, but comparable to or greater than similar studies (see Supplementary Table 1); nevertheless, parallel forms were used for long-term verbal memory while in visual memory tasks, the use of the same version could have produced a learning effect. Furthermore, even if the groups included in this study are homogenous for tumor grading and treatment strategy, our dataset did not allow for disentangling treatment (e.g., surgery-only compared to surgery plus radiotherapy) from tumor grade effects. All HGGs patients included in this study were treated with surgery in combination with radiotherapy and chemotherapy, and further studies may try to better consider how adjuvant therapies could affect these findings. To conclude, as with all correlational studies, our significant associations may not be causal. Given all these limitations and the relatively small group effects, further functional reorganization studies are needed before these results may be generalized at the individual level for cognitive outcome prediction, in particular the role of the DMN. Lastly, our study focused on the characterization of functional reorganization in association with cognition. Recent studies, however, suggest that it is important to also consider the reorganization of white matter structural connectivity (Griffis et al. 2019; Reber et al. 2021).

## Conclusions

We show evidence that clinical features such as the lateralization of a glioma, together with its grade, may have an impact on neural longitudinal reorganization and, consequently, on cognitive recovery after surgical resection. Specifically, we characterize the plastic reshaping of resilient functional pathways, such as connections within DMN. Our findings suggest an entirely novel role of DMN in supporting post-surgical cognitive recovery. Thus, on one hand, the results of this study provide new information for the prognostic prediction in terms of cognitive outcome after surgical resection of both LGGs and HGGs. On the other hand, even if the DMN role in post-surgical recovery needs further confirmation, we introduce the concept of a new pre-surgical planning approach that considers intrinsic hub networks important for the neurocognitive recovery.

## Supplementary Information

Below is the link to the electronic supplementary material.

Supplementary file1 (DOCX 810 KB)

## Data Availability

Data and materials supporting the results or analyses presented in this study are available upon request.
