# Supplementary material for: The role of the default mode network in longitudinal functional brain reorganization of brain gliomas
Source: Brain Struct Funct. 2022 Apr 23;227(9):2923–37. doi: 10.1007/s00429-022-02490-1 (PMC9653323; doi:10.1007/s00429-022-02490-1)
Supplement: Supplementary file 1 — Supplementary file1 (DOCX 810 KB) [file 429_2022_2490_MOESM1_ESM.docx]

**The role of the default mode network in longitudinal functional brain reorganization of brain gliomas**

Authors: Francesca Saviola^1,+^ MSc, Luca Zigiotto^2,+^ PsyD, Lisa Novello^1^ MSc, Domenico Zacà^1^ PhD, Luciano Annicchiarico^2^ MD, Francesco Corsini^2^ MD, Umberto Rozzanigo^3^ MD, Costanza Papagno^1,4^ MD, PhD, Jorge Jovicich^1,*^ PhD, Silvio Sarubbo^2,*^ MD, PhD

**^†^ Equal first-author contributions**

*** Equal last-author contributions**

Affiliations:

1. Center for Mind/Brain Sciences, University of Trento, Rovereto, Italy
2. Department of Emergency, Division of Neurosurgery, Structural and Functional Connectivity Lab Project, “S.Chiara” Hospital, Azienda Provinciale per I Servizi Sanitari Trento, Italy
3. Department of Radiology, Division of Neuroradiology, “S. Chiara” Hospital, Azienda Provinciale Per I Servizi Sanitari Trento, Italy
4. Department of Psychology, Milano-Bicocca University, Milano, Italy

Corresponding Author’s name and current institution:

Francesca Saviola^1^

Corso Bettini. 31 -38068 Rovereto (TN), Italy

Corresponding Author’s Email: [francesca.saviola@unitn.it](mailto:francesca.saviola@unitn.it)

**Key Words**: Functional connectivity; Gliomas; Hubs; Default mode network; Cognitive recovery

**Supplementary material**

1. ***Demographical, neurocognitive, and clinical information of the sample***

Supplementary Table 2 shows the distribution of the timepoints across the sample divided by tumor grade and lateralization.

- 1. *Neuropsychological tests*

Supplementary Table 3 reports the list of the neuropsychological assessments performed in this study by dividing the tests for the cognitive domain of interest(Caffarra, Vezzadini, Dieci, Zonato, & Venneri, 2002; Carlesimo et al., 1996; Gainotti, Marra, & Villa, 2001; Giovagnoli et al., 1996; Laiacona, Barbarotto, Trivelli, & Capitani, 1993; Novelli, Papagno, Capitani, Laiacona, & al, 1986; Orsini et al., 1987; Robinson, Shallice, Bozzali, & Cipolotti, 2012; SPINNLER & H, 1987).

- 1. *Baseline differences*

Regarding functional connectivity (FC) no differences were found at baseline between groups, either considering tumor lateralization (see Supplementary Table 8; within DMN FC_(right>left)_: t-value=-0.8, p-value=0.40; within FTPN FC_(right>left)_: t-value=0.\, p-value=0.94; within *hubs* FC_(right>left)_: t-value=-1.1, p-value=0.26; between *hubs* and *no-hubs* FC_(right>left)_: t-value=-0.4, p-value=0.70; within *no-hubs* FC_(right>left)_: t-value=0.7, p-value=0.95) or tumor grade (see Supplementary Table 7; within DMN FC_(LGG>HGG)_: t-value=1.3, p-value=0.20; within FTPN FC_(LGG>HGG)_: t-value=-1.0, p-value=0.32; within *hubs* FC_(LGG>HGG)_: t-value=-0.2, p-value=0.81; between *hubs* and *no-hubs* FC_(LGG>HGG)_: t-value=-0.7, p-value=0.52; within *no-hubs* FC_(LGG>HGG)_: t-value=0.6, p-value=0.52). Supplementary Table 4 shows the baseline neurocognitive profile of the sample divided by tumor grade in all the domains of interest.

Supplementary Table 5 shows the baseline neurocognitive profile of the sample divided by tumor lateralization.

Supplementary Table 11 displays the mean neurocognitive scores for each time point for each cognitive test and the respective percentage of patients with deficits in our cohort.

Supplementary Table 7 shows the baseline spatial connectomic profile of the sample divided by tumor grade in all the domains of interest,

Supplementary Table 8 shows the baseline spatial connectomic profile of the sample divided by tumor lateralization.

- 1. *Percentage variation of neuropsychological and spatial connectome measures*

Supplementary Tables 6 and Supplementary Table 9 show, respectively, percentage variation of neurocognitive profile scores and changes in the spatial connectomic profile while comparing the baseline time point (before surgical resection) (~ 1 year after surgical resection).

- 1. *Tumor anatomical location distribution*

Figure S2 displays the anatomical localization of the tumor for high- and low-grade gliomas, respectively.

1. ***Supplementary Materials and Methods***
   1. *Brain tumor masks*

For each patient a pre-surgical tumor mask was manually created by i) determining the tumor edges and the voxels belonging to the tumor mass using T2-weighted images, FLAIR or contrast-enhanced T1-weighted images; ii) then drawing in MRIcron (https://www.nitrc.org/projects/mricron) on each slice of the original 3D anatomical T1-weighted images or T2/FLAIR. Finally, by counting the number of voxels in the mask image, a measure of tumor volume before the surgical resection (Table I, cm3) was extracted for each patient.

- 1. *Images pre-processing*

An off-line quality check of the rs-fMRI data was performed before and after pre-processing steps, and no subject was discarded. Visual quality assurance on raw data included visual inspection of temporal Signal-to-noise ratio and standard deviation maps. Once pre-processing was performed motion parameters and power spectra of the time series were inspected together with co-registration to the T1-weighted anatomical scan and normalization to MNI steps.

Structural T1-weighted images and rs-fMRI time series were pre-processed with SPM12 software (<https://www.fil.ion.ucl.ac.uk/spm/software/download/>) as previously described in Zacà et al., 2018(Zacà et al., 2018). First, all images were converted from DICOM to Nifti, then for the rs-fMRI only, volumes included in the first 10 seconds of the acquisition were removed to allow the signal steady state. Pre-processing steps include: (1) Slice timing and head motion correction; (2) Co-registration of the T1-weighted image to the rs-fMRI time series; (3) T1-weighted image segmentation to grey matter, white matter, and cerebrospinal fluid; (4) rs-fMRI Temporal filtering: a) median, b) 4th order linear de-trending and c) 2nd order low pass filtering (Butterworth f<0.1 Hz); (5) Regression from the rs-fMRI time series of the 6 head motion parameters and white matter and cerebrospinal fluid signals; (7) Normalization to standard MNI template space; (8) Spatial smoothing with 2 voxels Full-Width Half Maximum Gaussian kernel size.

- 1. *Parcellation*
     1. *Parcellation strategies*

To verify the robustness of the results as a function of atlas parcellation strategies, and more specifically evaluate the use of a functional versus an anatomical atlas, the full analyses (definition of FC matrices and functional connectome analyses) was repeated using another parcellation atlas, the AAL atlas(Messé, 2020; Tzourio-Mazoyer et al., 2002) The spatial connectomic analysis resulted in no significant longitudinal changes (p-value>0.05) for all predictors of all linear mixed models in the case of AAL parcellation.

- - 1. *Tumor overlap with the parcellation*

Supplementary Table 10 represent the extent of overlap between the tumor mask computed at baseline and respectively the *hubs* and *no-hubs* selection with the two different parcellation strategies.

To be noticed for *no-hubs* regions selection the choice of the parcellation is strongly impacted in respect to the number of percentage voxels in common with the tumor tissues. This may be due to the nature of the parcellation which on one side is based on anatomical landmarks, (AAL(Tzourio-Mazoyer et al., 2002)) whereas on the other is jointly constructed based on well-known functional resting-state networks (Gordon(Gordon et al., 2016)).

- 1. *Linear mixed models*

The statistical evaluation of spatial connectomic measures, neuropsychological assessment, and the association of the two overtime across the sample of brain tumor patients, was performed by fitting separated linear mixed models for each metric.

For assessing the spatial connectomic profile and the neuropsychological profile, two different linear mixed models (i.e. one for cognitive profile and one for FC profile) were used with a fixed effect of *time*, a fixed effect of *time x tumor-grade interaction* (including two subgroups of patients: HGGs and LGGs) and a fixed effect of *time x tumor-lateralization interaction* (including two subgroups of patients divided by tumor hemisphere location, e.g. left-lateralized or right-lateralized). Random intercepts across the variables were used in the models to account for individual differences either in cognitive or FC profile. All models were adjusted for age, sex, preoperative tumor volume, WHO grading, and genetic profile. The output of the linear mixed model was reported as estimate standardized coefficient with corresponding p-value and effect size.

Furthermore, a final third linear mixed model was computed to test the association between cognitive outcome and FC: having the cognitive measures as a response, we assessed the significance of the fixed effect of interaction between *time x FC metrics*. In this latter model, the same random intercepts and all variables used in previous models were considered.

For all the three performed types of models, no assumption was made on the presence of all time points for every tested subject.

The linear mixed model for each spatial connectomic metric is presented here:

*[Spatial connectomic metric]* ~ *time × tumor grade + time × tumor lateralization + gender + age + tumor volume + tumor IDH mutation + tumor MGMT methylation + tumor staging + (1 | Subject)*

The linear mixed model for each neuropsychological measure is presented here:

*[Neuropsychological measure]* ~ *time × tumor grade + time × tumor lateralization + gender + age + tumor volume + tumor IDH mutation + tumor MGMT methylation + tumor staging + (1 | Subject)*

The linear mixed model for the association between each neuropsychological measure and spatial connectomic metrics is presented here:

*[Neuropsychological measure]* ~ *time × within DMN FC + time × within FTPN FC+ time × within no-hubs FC + time × between hubs and no-hubs FC + tumor grade + tumor lateralization + gender + age + tumor volume + tumor IDH mutation + tumor MGMT methylation + tumor staging + (1 | Subject)*

**Supplementary Table 1** Summary of studies evaluating how cognition and/or functional connectivity are affected by brain tumors. The study here presented (last row), is the first study of low- and high-grade brain tumors followed longitudinally after brain surgery with both resting-state functional connectivity and comprehensive cognitive evaluations. BT: Brain tumors patients; HC: healthy controls; HGG: high-grade glioma; LGG: low-grade glioma; CNS VS: Dutch Computerized Neuropsychological assessments (e.g. CNS Vital Signs, LLC, Morrisville, North Carolina, USA); WAIS-II: Wechsler adult intelligence score; IQ: Intelligence Quotient; WMS-R: Wechsler Memory Scale; MEG: Magnetoencephalography; Rs-fMRI: resting-state functional MRI; Task-based fMRI: task-based functional MRI; FC: functional connectivity

| **Studies** | **Experimental**  **Design** | **Patients** | **Tumor**  **treatment** | **Tumor lateralization (Left/Right)** | **Study**  **details** | **Main**  **findings** |
| --- | --- | --- | --- | --- | --- | --- |
| ***BEHAVIORAL TUMOR STUDIES*** | | | | | | |
| Correa et al., 2007(Correa et al., 2007) | Cross-sectional | 40 LGG | - 24 no treatment, - 11 radiotherapy, - 4 chemotherapy, - 1 radiotherapy and chemotherapy. | N.A. | - Within-subject design - Neuropsychological domains:   Attention, Executive, Verbal and Non-Verbal Memory, Psychomotor, Language, Visual Construction. | Radiotherapy and chemotherapy, disease duration, and antiepileptic treatment contributed to mild cognitive difficulties in LGG patients. |
| Correa et al., 2008(Correa et al., 2008) | Longitudinal | 25 LGG | - 16 no treatment, - 6 radiotherapy, - 3 chemotherapy | N.A. | - Within-subject design - 3 time points:   - before surgery,   - 6 months after surgery,   - 12 months after surgery. - Neuropsychological battery domains: (same as Correa et al., 2007) | 1. LGG longitudinal improvement in nonverbal Memory (no treatment difference); 2. LGG declined slightly by the 12-month evaluation. 3. Longitudinally phonemic verbal fluency, mood, and quality of life improved slightly but if treated with radiotherapy patients seen at longer intervals declined. |
| Satoer et al., 2013(Satoer, Vincent, Smits, Dirven, & Visch-Brink, 2013) | Longitudinal | 27 LGG,  17 HGG | - 16 only surgery, - 19 surgery and radiotherapy, - 4 surgery and chemotherapy, - 6 surgery and radiotherapy and chemotherapy | 42/3 | - Within-subject design - 3 time points:   - before surgery   - 3 months after surgery   - 12 months after surgery - Neuropsychological battery domains:   Attention, Executive, Language. | 1. Pre- and post-operatively, impairments were found in all cognitive domains (Language, Memory, Attention and Executive) 2. Post-operatively, permanent improvement observed in Memory, whereas deterioration was found in Language. 3. Between 3 months and 1 year, patients improved on Language. 4. There was no influence of tumor- or treatment-related factors on cognitive changes. |
| Habets et al., 2014(Habets et al., 2014) | Longitudinal | 62 HGG (pre),  39 HGG (post) | Only surgery | 28/34 | - Within-subject design - 2 time points:   - before surgery,   - weeks after surgery - Neuropsychological battery domains:   Verbal Memory, Working Memory, Executive, Psychomotor, Attention, Processing speed, Visuo-construction. | 1. Left hemisphere tumor localization was associated with worse verbal Memory, and larger tumors in this hemisphere with poorer Executive functioning. 2. Changes in cognitive performance at follow-up relative to baseline were unrelated to tumor characteristics. |
| Van Loenen et al., 2018(van Loenen et al., 2018) | Longitudinal | 125 HGG (pre),  84 HGG (post) | - 3 only surgery, - 8 surgery and radiotherapy, - 71 surgery and radiotherapy and chemotherapy | 45/80 | - Within-subject design - 2 time points:   - before surgery   - 3 months after surgery - Neuropsychological battery domains (CNS VS):   Verbal and Non-Verbal Memory,  Psychomotor speed, Processing speed,  Reaction Time, Complex attention,  Cognitive flexibility. | Lower performance of patients with Glioblastoma on all cognitive domains before and 3 months after surgery |
| Lahti et al., 2018(Lahti, Saunamäki, Salo, Niemelä, & Jehkonen, 2018) | Longitudinal | 31 HC,  18 LGG | - 3 only surgery, - 15 surgery and radiotherapy | N.A. | - Between-subject design - 3 time points:   - before surgery,   - 3 months after surgery,   - 12 months after surgery - Neuropsychological battery:   WAIS Similarities, WAIS Block design, Word list recall, Word list delayed recall, Story immediate recall, Story delayed recall, Stroop Time, Serial seven subtraction test | 1. The tumor patients with frontal and large tumors showed impairment virtually across all cognitive domains. 2. LGG group relative modest cognitive improvement. 3. Most of this improvement did not emerge until the 12 months follow-up. 4. Cognitive recovery after the surgery is more noticeable in patients with small tumors, and the recovery will require a minimum of one year timewise. |
| ***FUNCTIONAL TUMOR STUDIES*** | | | | | | |
| Briganti et al., 2012(Briganti et al., 2012) | Cross-sectional | 13 HC,  39 BT | none | 39/0 | - Between-subject design - Task fMRI | Left-hemisphere tumor reduced the degree of FC between language-related brain regions. |
| Esposito et al., 2012(Esposito et al., 2012) | Cross-sectional | 14 HC,  10 LGG,  14 HGG | None | 24/0 | - Between-subject design - Task fMRI | 1. Reduced DMN connectivity was detected in BT patients with respect to HC. 2. Modifications were closely related to tumor grading. 3. The DMN lateralized to the hemisphere contralateral to the tumor in LGG, but not in HGG patients. |
| Derks et al., 2017(Derks et al., 2017) | Cross-sectional | 19 HC,  41 LGG,  30 HGG | none | 40/31 | - Between-subject design - Task fMRI & rs-fMRI study | 1. Patients before surgery higher within no-Hubs connectivity and lower within Hubs connectivity compared to HC. 2. Patients before surgery had a significantly lower variance in FC compared to HC. |
| Touvinient et al., 2016(Tuovinen et al., 2016) | Longitudinal | 3 LGG | - 1 radiotherapy - 1 surgery and radiotherapy - 1 chemotherapy and radiotherapy | N.A. | - Within--subject design - 2 timepoints:   - before radiotherapy   - after radiotherapy | 1. If tumor lesion relates to a functional hub (DMN), these changes involve long-range connections. 2. In this case post-surgery + radiotherapy global but temporary improvement in FC. |
| Harris et al., 2014(Harris et al., 2014) | Longitudinal | 12 HC,  21 LGG,  47 HGG | - 21 surgery - 47 surgery and radiotherapy or chemotherapy | 68/0 | - Between-subject design - 2 timepoints:   - New glioma   - Recurrent gliomas | 1. DMN integrity was lower in HGG compared with LGG. 2. Tumors in the left parietal lobe showed a more impaired DMN compared with tumors in the frontal lobe, while tumors within and outside the network nodes did not differ significantly. 3. Higher tumor grade along with prior surgery and/or treatment causes the largest reduction in DMN functional connectivity in patients with primary gliomas, and that tumor location has an impact on connectivity. |
| ***MULTIMODAL FUNCTIONAL AND BEHAVIORAL TUOR STUDIES*** | | | | | | |
| Derks et al., 2019(Derks et al., 2019) | Cross-sectional | 28 LGG,  26 HGG | none | 30/22  (2 bilateral) | - Within-subject design - MEG study | 1. HGG IDH-mutated tumors result in poorer cognitive performance in Verbal Memory 2. Global functional connectivity is lower in patients without IDH mutation. 3. Having lower functional alpha connectivity relates to poorer cognitive performance in patients. |
| De Baene et al., 2019(De Baene, Rutten, & Sitskoorn, 2019) | Cross-sectional | 29 LGG,  16 HGG | none | 45/0 | - Within-subject design - rs-fMRI study - Neuropsychological domains (CNS VS):   - Verbal and Non-Verbal Memory, Psychomotor speed, Processing speed, Reaction Time, Complex attention, Cognitive flexibility. | 1. Better complex attention and cognitive flexibility performance are achieved with a better spread of information over the contralesional hemisphere through mutually interconnected contralesional hubs. 2. Need to recognize the functional contribution of remote, undamaged regions and to focus more on the graph metrics of the contralesional hemisphere in the search for predictors of cognitive functioning in patients with brain tumor. |
| Xu et al., 2013(Xu et al., 2013) | Cross-sectional | 20 HC,  21 LGG | none | 12/9 | - Between-subject design - rs-fMRI study - Neuropsychological battery:   - WAIS-III-RC: Verbal IQ; Performance IQ; Comprehension; Perceptual organization; Working Memory | 1. Compared with controls, LGG patients display disturbed small-world manner and decreased global efficiency. 2. Global efficiency correlated positively with IQ test scores in LGG patients. 3. Network hubs in LGG group are altered compared to that of the control group |
| Maesawa et al., 2015(Maesawa et al., 2015) | Cross-sectional | 12 HC,  7 LGG,  5 HGG | none | 12/0 | - Between-subject design - rs-fMRI study - Neuropsychological battery: - WAIS-III: Verbal IQ; Performance IQ Full IQ; Verbal comprehension; Perceptual organization; Working memory - WMS-R: Verbal Memory; Visual Memory; Memory; Attention; Delayed recall | 1. Left-hemisphere tumor decrease FC in DMN relative to HC 2. Left-hemisphere tumors showed cross-sectional connectivity changes in DMN correlated with Attention, Working Memory, Verbal and full IQ scale. |
| van Dokkum et al., 2019(van Dokkum et al., 2019) | Cross-sectional | 19 HC,  39 LGG | 39 surgery | 39/0 | - Between-subject design - rs-fMRI - Neuropsychological performance:   - Picture naming | 1. Picture naming was dependent on the semantic network (integration and interaction of regions within multiple resting-state brain networks) |
| This study | Longitudinal | 11 LGG,  17 HGG | - 11 surgery - 17 surgery and radiotherapy | 15/13 | - Within-subject design - Neuropsychological battery   - Language, Executive, Memory, Attention - Rs-fMRI study, variable time points:   - before surgery,   - 1-3 months after surgery,   - 6-9 months after surgery,   - 12-15 months after surgery. |  |

**Supplementary Table 2** Distribution of longitudinal time points in the dataset divided by the stratified tumor group. Since the timepoints were not acquired at recurrent and stable time differences, patients are grouped into timepoints (T) to better understand their longitudinal distribution. T_0_ : baseline pre-surgical timepoint (0 months), T_1_ : first time point after surgery (1-4 months), T_2_: second timepoint after surgery (5-7 months), T_3_: third timepoint after surgery (8-10 months), T_4_: fourth time point after surgery (11-15 months)

|  | ***Brain tumor types*** | | |
| --- | --- | --- | --- |
|  | *low-grade gliomas* | *high-grade gliomas* | ***TOTAL*** |
| ***Total*** | 30 | 43 | 73 |
| ***Baseline T_0_***  *(# patients)* | 11 | 17 | 28 |
| ***T_1_***  *(# patients, mean months*± *SD)* | 8, 3 ± 0 | 7, 2 ± 1 | 3.3 ± 0.9 |
| ***T_2_***  *(# patients, mean months*± *SD)* | 5, 6 ± 1 | 7, 6 ± 1 | 6.2 ± 0.9 |
| ***T_3_***  *(# patients, mean months*±*, SD)* | 4, 9 ± 1 | 6, 9 ± 1 | 8.9 ± 1.0 |
| ***T_4_***  *(# patients, mean months*± *SD)* | 2, 13 ± 2 | 6, 12 ± 1 | 12.1 ± 1.3 |

**Supplementary Table 3** List of the neuropsychological tests grouped by cognitive domain.

| **Cognitive Domain** |  |
| --- | --- |
|  | *Neuropsychological tests* |
| ***Memory*** | *Digit span*  *Corsi span*  *15 Rey’s word list immediate recall*  *15 Rey’s word list delayed recall (parallel)*  *Rey complex figure delayed reproduction* |
| ***Attention*** | *Attentional Matrices*  *Trial Making Test a*  *Trial Making Test b* |
| ***Language*** | *Picture naming test*  *Semantic fluency* |
| ***Constructional praxis*** | *Rey complex figure copy* |
| ***Executive functions*** | *Phonemic fluency*  *Trial making Test b-a* |

**Supplementary Table 4** Neurocognitive profile at baseline divided by tumor grade. The table reports for each neuropsychological score, grouped by cognitive domain, its range and relevant cut-off scores, and the baseline scores, mean and standard deviation, for the two tumor grade groups (high-grade and low-grade gliomas).

|  |  | ***Brain tumor types*** | | |  |  |
| --- | --- | --- | --- | --- | --- | --- |
|  | *range, cut-off* | *low-grade gliomas* | *high-grade gliomas* | ***TOTAL*** | ***t-statistic*** | ***p-value*** |
| ***Patients*** |  | 11 | 17 | 28 | NA | NA |
| ***Memory***  *Digit span*  *Corsi span*  *15 Rey’s word list immediate recall*  *15 Rey’s word list delayed recall*  *Rey complex figure delayed reproduction* | [0-9], < 3.75  [0-9], < 3.50  [0-75], < 28.53  [0-15], <4.69  [0-36], < 9.47 | 5.1 ± 1.2  4.7 ± 0.8  41.0 ± 8.2  7.3 ± 3.4  14.3 ± 3.8 | 5.0 ± 1.0  4.9 ± 1.2  36.0 ± 7.2  6.4 ± 2.0  12.5 ± 6.2 | 5.0 ± 1.0  4.8 ± 1.2  38.0 ± 7.7  6.7 ± 2.0  13.2 ± 5.7 | 0.29  -0.73  1.63  0.78  0.86 | 0.77  0.46  0.11  0.44  0.39 |
| ***Attention***  *Attentional Matrices*  *Trial Making Test a*  *Trial Making Test b* | [0-60], < 30  [0-∞], >93  [0-∞], >282 | 46.5 ± 5.7  30.9 ± 13.6  86.2 ± 36.8 | 42.7 ± 10.4  29.2 ± 17.6  101.0 ± 51.3 | 44.2 ± 9.4  29.9 ± 16.9  95.2 ± 49.1 | 1.02  0.26  -0.82 | 0.31  0.79  0.41 |
| ***Language***  *Picture naming test*  *Semantic fluency* | [0-80], < 60  [0-∞], <25 | 74.4 ± 4.1  43.1 ± 5.6 | 72.3 ± 11.4  39.7± 11.0 | 73.10 ± 10.0  41.1 ± 10.1 | 0.57  0.94 | 0.57  0.35 |
| ***Constructional praxis***  *Rey complex figure copy* | [0-36], < 28.88 | 32.6 ± 3.4 | 32.7 ± 4.2 | 32.7 ± 4.0 | -0.12 | 0.90 |
| ***Executive functions***  *Phonemic fluency*  *Trial making Test b-a* | [0-∞], <17  [0-∞], >187 | 31.1 ± 7.8  56.5 ± 26.5 | 28.4 ± 7.7  72.7 ± 36.9 | 29.4 ± 7.8  66.3 ± 35.9 | 0.87  -1.25 | 0.39  0.22 |

**Supplementary Table 5** Neurocognitive profile at baseline divided by tumor hemispheric lateralization. This table reports for each neuropsychological score, grouped by cognitive domain, its range and relevant cut-off scores and the baseline scores, mean and standard deviation, for the two hemispheric lateralization groups (left- and right-hemisphere gliomas).

|  |  | ***Brain tumor hemispheric lateralization*** | | |  |  |
| --- | --- | --- | --- | --- | --- | --- |
|  | *range, cut-off* | *left-hemisphere gliomas* | *right-hemisphere gliomas* | ***TOTAL*** | ***Statistic*** | ***p-value*** |
| ***Patients*** |  | 15 | 13 | 28 | NA | NA |
| ***Memory***  *Digit span*  *Corsi span*  *15 Rey’s word list immediate recall*  *15 Rey’s word list delayed recall*  *Rey complex figure delayed reproduction* | [0-9], < 3.75  [0-9], < 3.50  [0-75], < 28.53  [0-15], <4.69  [0-36], < 9.47 | 4.9 ± 1.1  4.8 ± 1.1  36.8 ± 8.6  7.1 ± 3.2  13.7 ± 5.2 | 5.2 ± 1.1  4.9 ± 1.2  39.4 ± 7.0  6.4 ± 1.9  12.7 ± 5.7 | 5.0 ± 1.0  4.8 ± 1.2  38.0 ± 7.7  6.7 ± 2.0  13.2 ± 5.7 | -0.42  -0.53  0.49  -0.93  0.70 | 0.67  0.60  0.62  0.35  0.48 |
| ***Attention***  *Attentional Matrices*  *Trial Making Test a*  *Trial Making Test b* | [0-60], < 30  [0-∞], >93  [0-∞], >282 | 46.0 ± 9.1  332.4 ± 18.0  102.5 ± 54.4 | 42.1 ± 8.5  26.9 ± 13.0  86.8 ± 34.2 | 44.2 ± 9.4  29.9 ± 16.9  95.2 ± 49.1 | 1.18  0.91  0.89 | 0.24  0.36  0.33 |
| ***Language***  *Picture naming test*  *Semantic fluency* | [0-80], < 60  [0-∞], <25 | 71.3 ± 11.7  39.4 ± 5.8 | 75.2 ± 4.4  42.9 ± 12.1 | 73.10 ± 10.0  41.1 ± 10.1 | -1.14  -1.00 | 0.26  0.32 |
| ***Constructional praxis***  *Rey complex figure copy* | [0-36], < 28.88 | 31.9 ± 3.8 | 33.5 ± 3.8 | 32.7 ± 4.0 | -1.13 | 0.26 |
| ***Executive functions***  *Phonemic fluency*  *Trial making Test b-a* | [0-∞], <17  [0-∞], >187 | 30.1 ± 5.8  70.2 ± 37.8 | 28.7 ± 9.6  61.8 ± 29.0 | 29.4 ± 7.8  66.3 ± 35.9 | 0.46  0.65 | 0.64  0.51 |

**Supplementary Table 6** Mean percentage changes of neurocognitive profile scores while comparing baseline (before surgical resection, T_0_) to the last time point for each patient (~ 1 year after surgical resection, T_3_ or T_4_)

|  | ***Brain tumor types*** | | |  |
| --- | --- | --- | --- | --- |
|  | *left-hemisphere gliomas* | *right-hemisphere gliomas* | *low-grade gliomas* | *high-grade gliomas* |
| ***Patients*** | 15 | 13 | 11 | 17 |
| ***Memory***  *Digit span*  *Corsi span*  *15 Rey’s word list immediate recall*  *15 Rey’s word list delayed recall*  *Rey complex figure delayed reproduction* | 3.2 ± 8.1  -2.6 ± 9.8  -0.6 ± 12.5  -11.1± 19.4  15.5 ± 18.0 | -1.4 ± 13.8  0.2 ± 12.6  -4.1 ± 9.9  2.2 ± 19.8  16.3 ± 21.3 | 2.5 ± 8.9  1.5 ± 10.8  -1.2 ± 11.7  -5.7 ± 22.3  14.8 ± 19.4 | 0.0 ± 12.6  -3.3 ± 11.1  -3.2 ± 10.7  -3.9 ± 19.6  13.3 ± 19.9 |
| ***Attention***  *Attentional Matrices*  *Trial Making Test a*  *Trial Making Test b* | -3.1 ± 13.1  7.6 ± 18.1  3.8 ± 43.3 | 3.5 ± 15.5  5.5 ± 11.1  0.1 ± 42.7 | -0.5 ± 14.85  -0.3 ± 12.4  -17.75 ± 25.4 | 0.5 ± 14.6  11.5 ± 16.1  17.2 ± 46.6 |
| ***Language***  *Picture naming test*  *Semantic fluency* | 1.8 ± 3.2  -0.6 ± 9.7 | 2.1 ± 4.7  1.8 ± 7.5 | 1.7 ± 3.8  3.2 ± 6.7 | 2.1 ± 4.1  -1.3 ± 9.5 |
| ***Constructional praxis***  *Rey complex figure copy* | -0.6 ± 9.7 | -3.9 ± 7.8 | -2.1 ± 9.9 | -4.7 ± 12.1 |
| ***Executive functions***  *Phonemic fluency*  *Trial making Test b-a* | 3.1 ± 13.6  -2.8 ± 32.2 | 5.0 ± 10.1  -4.4 ± 37.7 | 5.7 ± 9.7  -12.7 ± 22.0 | 2.9 ± 13.2  3.3 ± 39.9 |

**Supplementary Table 7** Spatial connectomic profile at baseline divided by tumor grade. This table reports for each spatial connectomic measure, grouped by within or between networks connectivity, the mean functional connectivity, and standard deviation, for the grade groups (low- and high-grade gliomas).

|  | ***Brain tumor types*** | | |  |  |
| --- | --- | --- | --- | --- | --- |
|  | *low-grade gliomas* | *high-grade gliomas* | ***TOTAL*** | ***Statistic*** | ***p-value*** |
| ***Patients*** | 11 | 17 | 28 | NA | NA |
| ***within network FC***  *within hubs*  *within DMN*  *within FTPN*  *within no-hubs* | 1.1 ± 0.0  1.1 ± 0.1  1.1 ± 0.1  1.0± 0.0 | 1.1± 0.1  1.1 ±0.1  1.2 ± 0.1  1.0± 0.0 | 1.1 ± 0.1  1.1 ± 0.1  1.2 ± 0.1  1.0 ± 0.0 | t(26)=0.24  t(26)=-1.02  t(26)=1.31  t(26)=0.64 | 0.81  0.20  0.32  0.52 |
| ***between network FC***  *between hubs and no-hubs* | 0.9 ± 0.0 | 0.9 ± 0.0 | 0.9 ± 0.0 | t(26)=-0.65 | 0.52 |

**Supplementary Table 8** Spatial connectomic profile at baseline divided by tumor hemispheric lateralization. The table reports for each spatial connectomic measure, grouped by within or between networks connectivity, the mean functional connectivity, and standard deviation, for the hemispheric lateralization groups (left- and right-lateralized gliomas).

|  | ***Brain tumor hemispheric lateralization*** | | |  |  |
| --- | --- | --- | --- | --- | --- |
|  | *left-hemisphere gliomas* | *right-hemisphere gliomas* | ***TOTAL*** | ***Statistic*** | ***p-value*** |
| ***Patients*** | 15 | 13 | 28 | NA | NA |
| ***within network FC***  *within hubs*  *within DMN*  *within FTPN*  *within no-hubs* | 1.1 ± 0.1  1.1 ± 0.1  1.1 ± 0.1  1.0 ± 0.0 | 1.0± 0.1  1.1 ± 0.1  1.1 ± 0.1  1.0± 0.0 | 1.1 ± 0.1  1.1 ± 0.1  1.2 ± 0.1  1.0 ± 0.0 | t(26)=1.1  t(26)=0.84  t(26)=-0.1  t(26)=-0.7 | 0.26  0.40  0.94  0.70 |
| ***between network FC***  *between hubs and no-hubs* | 1.0 ± 0.0 | 1.0 ± 0.0 | 0.9 ± 0.0 | t(26)=0.4 | 0.48 |

**Supplementary Table 9** Mean relative changes of spatial connectomic profile scores while comparing baseline (before surgical resection, T_0_) to the last time point for each patient (~ 1 year after surgical resection, T_3_ or T_4_)

|  | ***Brain tumor types*** | | |  |
| --- | --- | --- | --- | --- |
|  | *left-hemisphere gliomas* | *right-hemisphere gliomas* | *low-grade gliomas* | *high-grade gliomas* |
| ***Patients*** | 15 | 13 | 11 | 17 |
| ***within network FC***  *within hubs*  *within DMN*  *within FTPN*  *within no-hubs* | 0.01 ± 0.08  0.03± 0.12  0.02± 0.17  -0.01± 0.02 | 0.04 ± 0.09  0.03± 0.20  -0.03 ±0.19  -0.01± 0.02 | 0.01± 0.01  0.03± 0.16  -0.03 ± 0.18  -0.01± 0.01 | 0.03± 0.01  0.02± 0.16  0.01 ± 0.18  -0.01± 0.02 |
| ***between network FC***  *between hubs and no-hubs* | -0.00 ± 0.03 | 0.02± 0.04 | 0.01 ± 0.03 | 0.01 ± 0.04 |

**Supplementary Table 10** Percentage of tumor mask voxels overlapping with the *hubs* and *no-hubs* functional networks relative to the total amount of voxels belonging to the network. Abbreviations: DMN, Default Mode Network; FTPN, Frontoparietal Network; AAL2, Automatic Anatomical Labelling Atlas.

|  | ***Brain tumor types*** | | |  |
| --- | --- | --- | --- | --- |
|  | *left-hemisphere gliomas* | *right-hemisphere gliomas* | *low-grade gliomas* | *high-grade gliomas* |
| ***Patients*** | 15 | 13 | 11 | 17 |
| ***Gordon parcellation***  *% hubs overlap*  *% no-hubs overlap* | 1.3 ± 1.8  2.5 ± 2.2 | 1.3 ± 1.8  2.5 ± 2.2 | 1.1± 1.9  1.1 ± 1.0 | 2.0± 2.4  2.6 ± 2.1 |
| *% DMN overlap*  *% FTPN overlap* | 1.6 ± 2.6  1.2 ± 2.0 | 1.7 ± 2.5  1.5 ± 2.1 | 0.8± 1.6  1.1 ± 2.0 | 2.0± 2.7  1.9 ± 2.3 |
| ***AAL2 parcellation***  *% hubs overlap*  *% no-hubs overlap* | 0.6 ± 1.0  9.9 ± 14.1 | 1.2 ± 1.0  17.7 ± 15.5 | 0.5 ± 0.7  8.8 ± 8.7 | 1.3 ± 1.1  18.9 ±17.6 |

**Supplementary Table 11** Anatomical distribution of tumor by brain lobes in the dataset divided by the stratified tumor group.

|  | ***Brain tumor types*** | | |
| --- | --- | --- | --- |
|  | *low-grade gliomas* | *high-grade gliomas* | ***TOTAL*** |
|  | 11 | 17 | 28 |
| ***Temporal lobe*** | 4 | 2 | 6 |
| ***Temporo-parietal lobe*** | / | 1 | 1 |
| ***Frontal lobe*** | 2 | 7 | 9 |
| ***Cingulum*** | 1 | / | 1 |
| ***Fronto-parietal lobe*** | / | 1 | 1 |
| ***Fronto-temporal lobe*** | / | 2 | 2 |
| ***Insula*** | / | 1 | 1 |
| ***Occipital lobe*** | 1 | 1 | 2 |
| ***Parietal lobe*** | 2 | 3 | 5 |

**Supplementary Table 12** Mean neurocognitive profile scores for each cognitive test at the different time-points and percentage of patients under the cut-offs.

| **Test** | **Time of assessment** | **N** | **Mean scores (SD)** | **% of deficit** |
| --- | --- | --- | --- | --- |
| Digit span | T_0_ (baseline) | 28 | 5.03 ($\pm$ 1.08) | 10.71% |
|  | T_1_ (1-4 months) | 15 | 5.12 ($\pm$ 0.89) | 0% |
|  | T_2_ (5-7 months) | 12 | 5.33 ($\pm$ 1.29) | 8.33% |
|  | T_3_ (8-10 months) | 10 | 5.48 ($\pm$ 1.49) | 10% |
|  | T_4_ (11-15 months) | 8 | 4.91 ($\pm$ 0.93) | 0% |
| Corsi span | T_0_ (baseline) | 28 | 4.86 ($\pm$ 1.11) | 7.14% |
|  | T_1_ (1-4 months) | 15 | 4.95 ($\pm$ 0.99) | 0% |
|  | T_2_ (5-7 months) | 12 | 5 ($\pm$ 1.11) | 0% |
|  | T_3_ (8-10 months) | 10 | 4.88 ($\pm$ 0.89) | 0% |
|  | T_4_ (11-15 months) | 8 | 5 ($\pm$ 1.4) | 12.5% |
| 15 Rey’s word list: immediate recall | T_0_ (baseline) | 28 | 38.02 ($\pm$ 7.86) | 10.71% |
|  | T_1_ (1-4 months) | 15 | 37.82 ($\pm$ 7.7) | 0% |
|  | T_2_ (5-7 months) | 12 | 38.36 ($\pm$ 7.43) | 8.33% |
|  | T_3_ (8-10 months) | 10 | 40.73 ($\pm$ 7.98) | 10% |
|  | T_4_ (11-15 months) | 8 | 34.39 ($\pm$ 6.25) | 12.5% |
| 15 Rey’s word list: delayed recall | T_0_ (baseline) | 28 | 6.76 ($\pm$ 2.67) | 21.43% |
|  | T_1_ (1-4 months) | 15 | 5.88 ($\pm$ 2.24) | 53.33% |
|  | T_2_ (5-7 months) | 12 | 6.72 ($\pm$ 2.01) | 8.33% |
|  | T_3_ (8-10 months) | 10 | 7.01 ($\pm$ 3.3) | 20% |
|  | T_4_ (11-15 months) | 8 | 6.64 ($\pm$ 1.57) | 25% |
| Rey’s complex figure: delayed reproduction | T_0_ (baseline) | 28 | 13.23 ($\pm$ 5.36) | 21.43% |
|  | T_1_ (1-4 months) | 15 | 18.84 ($\pm$ 6.45) | 6.67% |
|  | T_2_ (5-7 months) | 12 | 18.55 ($\pm$ 5.43) | 0% |
|  | T_3_ (8-10 months) | 10 | 17.85 ($\pm$ 8.2) | 10% |
|  | T_4_ (11-15 months) | 8 | 26 ($\pm$ 5.8) | 0% |
| Attentional matrices | T_0_ (baseline) | 28 | 44.19 ($\pm$ 8.9) | 10.71% |
|  | T_1_ (1-4 months) | 15 | 47.02 ($\pm$ 7.88) | 0% |
|  | T_2_ (5-7 months) | 12 | 44.81 ($\pm$ 6.41) | 0% |
|  | T_3_ (8-10 months) | 10 | 46.23 ($\pm$ 9.89) | 10% |
|  | T_4_ (11-15 months) | 8 | 44.84 ($\pm$ 6.01) | 0% |
| Trial Making Test a | T_0_ (baseline) | 28 | 29.86 ($\pm$ 15.89) | 0% |
|  | T_1_ (1-4 months) | 15 | 33.32 ($\pm$ 17.55) | 0% |
|  | T_2_ (5-7 months) | 12 | 33.47 ($\pm$ 13.97) | 0% |
|  | T_3_ (8-10 months) | 10 | 33.5 ($\pm$ 15.31) | 0% |
|  | T_4_ (11-15 months) | 8 | 31 ($\pm$ 18.17) | 0% |
| Trial Making Test b | T_0_ (baseline) | 28 | 95.18 ($\pm$ 46.03) | 0% |
|  | T_1_ (1-4 months) | 15 | 101.97 ($\pm$ 43.32) | 0% |
|  | T_2_ (5-7 months) | 12 | 100.04 ($\pm$ 46.39) | 0% |
|  | T_3_ (8-10 months) | 10 | 74.6 ($\pm$ 42.38) | 0% |
|  | T_4_ (11-15 months) | 8 | 96.25 ($\pm$ 71.84) | 0% |
| Picture naming test | T_0_ (baseline) | 28 | 73.11 ($\pm$ 9.18) | 3.57% |
|  | T_1_ (1-4 months) | 15 | 76.47 ($\pm$ 3.46) | 0% |
|  | T_2_ (5-7 months) | 12 | 71.33 ($\pm$ 12.9) | 8.33% |
|  | T_3_ (8-10 months) | 10 | 76.9 ($\pm$ 2.96) | 0% |
|  | T_4_ (11-15 months) | 8 | 76.13 ($\pm$ 5.41) | 0% |
| Semantic fluency | T_0_ (baseline) | 28 | 41.06 ($\pm$ 9.28) | 7.14% |
|  | T_1_ (1-4 months) | 15 | 39.42 ($\pm$ 7.29) | 0% |
|  | T_2_ (5-7 months) | 12 | 38.53 ($\pm$ 8.73) | 0% |
|  | T_3_ (8-10 months) | 10 | 45.5 ($\pm$ 12.83) | 0% |
|  | T_4_ (11-15 months) | 8 | 42.75 ($\pm$ 16.63) | 12.5% |
| Rey’s complex figure: copy | T_0_ (baseline) | 28 | 32.64 ($\pm$ 3.81) | 14.29% |
|  | T_1_ (1-4 months) | 15 | 32.76 ($\pm$ 3.77) | 13.33% |
|  | T_2_ (5-7 months) | 12 | 29.5 ($\pm$ 3.94) | 25% |
|  | T_3_ (8-10 months) | 10 | 31.55 ($\pm$ 2.86) | 10% |
|  | T_4_ (11-15 months) | 8 | 33.38 ($\pm$ 2.3) | 0% |
| Phonemic fluency | T_0_ (baseline) | 28 | 29.42 ($\pm$ 7.69) | 7.14% |
|  | T_1_ (1-4 months) | 15 | 32.46 ($\pm$ 12.48) | 0% |
|  | T_2_ (5-7 months) | 12 | 31.36 ($\pm$ 9.97) | 8.33% |
|  | T_3_ (8-10 months) | 10 | 31.75 ($\pm$ 11.94) | 10% |
|  | T_4_ (11-15 months) | 8 | 32.41 ($\pm$ 13.5) | 12.5% |
| Trial Making Test  b-a | T_0_ (baseline) | 28 | 66.29 ($\pm$ 33.66) | 0% |
|  | T_1_ (1-4 months) | 15 | 70.28 ($\pm$ 35.28) | 0% |
|  | T_2_ (5-7 months) | 12 | 67.5 ($\pm$ 38.6) | 0% |
|  | T_3_ (8-10 months) | 10 | 41.3 ($\pm$ 32.61) | 0% |
|  | T_4_ (11-15 months) | 8 | 65.25 ($\pm$ 56.18) | 0% |

**Supplementary Table 13** Anatomical locations for areas defined as belonging to the Default mode network

| ***Parcel*** | ***Hemisphere*** | ***Surface area (mm^2^)*** | ***Centroid (MNI)*** | ***Community*** | ***Harvard Oxford Label*** |
| --- | --- | --- | --- | --- | --- |
| 1 | L | 1411.6436 | -11.2 -52.4 36.5 | Default | Precuneus Cortex,  Cingulate gyrus |
| 4 | L | 368.6362 | -11.7 26.7 57 | Default | Superior Frontal gyrus |
| 6 | L | 1083.6667 | -47.2 -58 30.8 | Default | Angular gyrus,  Lateral occipital cortex |
| 25 | L | 200.8502 | -5.6 42.2 35.1 | Default | Superior Frontal gyrus, Paracingulate gyrus |
| 26 | L | 121.3918 | -1.7 -17.7 39.1 | Default | Cingulate gyrus |
| 44 | L | 518.7352 | -19.5 30.1 45.5 | Default | Superior Frontal gyrus |
| 94 | L | 485.8351 | -39.3 -73.9 38.3 | Default | Lateral Occipital cortex |
| 114 | L | 85.7529 | -27.5 53.6 0 | Default | Frontal pole |
| 116 | L | 539.1494 | -5.9 54.8 -11.3 | Default | Frontal medial cortex,  Frontal pole |
| 117 | L | 307.5771 | -6.8 38.2 -9.4 | Default | Paracingulate gyrus,  Cingulate gyrus |
| 126 | L | 141.899 | -63.2 -28.7 -7.2 | Default | Middle temporal gyrus,  Superior temporal gyrus |
| 127 | L | 776.4789 | -53.1 -11.4 -16 | Default | Middle temporal gyrus |
| 145 | L | 177.8342 | -15.9 48.6 37.2 | Default | Frontal pole |
| 146 | L | 272.8985 | -19.5 56.3 27.5 | Default | Frontal pole |
| 150 | L | 685.0619 | -6.5 54.7 18.1 | Default | Superior frontal gyrus, Paracingulate gyrus |
| 151 | L | 150.8711 | -15.7 64.7 13.7 | Default | Frontal pole |
| 152 | L | 176.6896 | -6 44.9 6.3 | Default | Cingulate gyrus,  Paracingulate gyrus |
| 154 | L | 104.9614 | -26.2 26.6 38.8 | Default | Middle frontal gyrus,  Superior frontal gyrus |
| 156 | L | 139.2005 | -29.3 16.8 50.7 | Default | Middle frontal gyrus,  Superior frontal gyrus |
| 157 | L | 351.7286 | -41.7 16.1 47.5 | Default | Middle frontal gyrus |
| 162 | R | 1286.0436 | 12.3 -51.6 34.5 | Default | Cingulate gyrus,  Precuneous cortex |
| 165 | R | 445.2963 | 11.9 21.9 59.9 | Default | Superior frontal gyrus |
| 184 | R | 375.3193 | 7.7 44.1 5.5 | Default | Cingulate gyrus,  Paracingulate gyrus |
| 186 | R | 85.9457 | 3 -19.6 37.9 | Default | Cingulate gyrus |
| 200 | R | 368.354 | 21.9 21 46.2 | Default | Superior frontal gyrus,  Middle frontal gyrus |
| 220 | R | 658.6387 | 48.9 -53 28.6 | Default | Angular gyrus |
| 225 | R | 259.4568 | 62.5 -25.6 -5.5 | Default | Middle temporal gyrus,  Superior temporal gyrus |
| 257 | R | 53.1597 | 7.4 -69.3 49.9 | Default | Precuneus cortex |
| 259 | R | 709.9593 | 46.5 -67.3 36.2 | Default | Lateral occipital cortex |
| 278 | R | 279.6353 | 4.8 65.1 -7.1 | Default | Frontal pole |
| 279 | R | 422.7278 | 7.2 48.4 -10.1 | Default | Frontal medial cortex, Paracingulate gyrus |
| 290 | R | 711.4259 | 57.5 -7.4 -16.4 | Default | Middle temporal gyrus |
| 315 | R | 200.1364 | 21 32.8 42.1 | Default | Superior Frontal gyrus,  Middle frontal gyrus |
| 316 | R | 111.1649 | 21.4 42.8 35.1 | Default | Frontal pole,  Superior frontal gyrus |
| 321 | R | 285.4723 | 16 61 19.8 | Default | Frontal pole |
| 322 | R | 494.1675 | 8.2 53.8 14 | Default | Paracingulate gyrus,  Frontal pole |
| 323 | R | 118.0971 | 5.9 54.9 29.4 | Default | Superior Frontal gyrus,  Frontal pole |
| 324 | R | 201.5247 | 13.8 46.7 42.1 | Default | Frontal pole |
| 325 | R | 251.6252 | 6.8 44.5 34.8 | Default | Superior frontal gyrus, Paracingulate gyrus |
| 326 | R | 83.9548 | 30.6 18.9 48.7 | Default | Middle frontal gyrus,  Superior frontal gyrus |
| 331 | R | 101.3923 | 54.4 1.1 -12.9 | Default | Superior temporal gyrus,  Middle temporal gyrus |

**Supplementary Table 14** Anatomical locations for areas defined as belonging to the Fronto-parietal network

| ***Parcel*** | ***Hemisphere*** | ***Surface area (mm^2^)*** | ***Centroid (MNI)*** | ***Community*** | ***Harvard Oxford Label*** |
| --- | --- | --- | --- | --- | --- |
| 7 | L | 91.3224 | -38.1 48.8 10.5 | FrontoParietal | Frontal pole |
| 9 | L | 95.001 | -55.9 -47.7 -9.3 | FrontoParietal | Middle temporal gyrus,  Inferior temporal gyrus |
| 24 | L | 281.9111 | -5.5 29.3 44 | FrontoParietal | Superior Frontal gyrus, Paracingulate gyrus |
| 78 | L | 388.3207 | -40.3 50.4 -4.8 | FrontoParietal | Frontal pole |
| 96 | L | 194.9111 | -34.1 -61 42.4 | FrontoParietal | Lateral Occipital cortex,  Angular gyrus |
| 108 | L | 86.8292 | -43 19.4 33.5 | FrontoParietal | Middle frontal gyrus,  Inferior frontal gyrus |
| 109 | L | 275.3985 | -40.2 23.6 23.3 | FrontoParietal | Middle frontal gyrus,  Inferior frontal gyrus |
| 148 | L | 193.79 | -21.3 63.1 1.9 | FrontoParietal | Frontal pole |
| 149 | L | 106.1582 | -28.6 50.9 10.1 | FrontoParietal | Frontal pole |
| 167 | R | 154.2867 | 47.9 -42.5 41.5 | FrontoParietal | Supramarginal gyrus,  Angular gyrus |
| 168 | R | 156.6639 | 38.1 45.9 7.7 | FrontoParietal | Frontal pole |
| 170 | R | 259.3776 | 59.7 -41 -10.9 | FrontoParietal | Supramarginal gyrus,  Middle temporal gyrus |
| 182 | R | 259.7731 | 7 25.7 47.3 | FrontoParietal | Paracingulate gyrus,  Superior frontal gyrus |
| 240 | R | 306.7999 | 42.8 48.3 -5.1 | FrontoParietal | Frontal pole |
| 260 | R | 59.9758 | 41.5 -53.5 44 | FrontoParietal | Angular gyrus,  Superior parietal lobule |
| 261 | R | 39.9006 | 35.7 -56.7 45.2 | FrontoParietal | Angular gyrus,  Superior parietal lobule |
| 272 | R | 168.7146 | 37.8 28.7 35.6 | FrontoParietal | Middle frontal gyrus |
| 273 | R | 356.9694 | 41.8 29.1 21.6 | FrontoParietal | Middle frontal,  Inferior frontal gyrus |
| 276 | R | 58.8152 | 38.6 18.8 25.5 | FrontoParietal | Middle frontal,  Inferior frontal gyrus |
| 277 | R | 327.9747 | 28.4 57 -5.1 | FrontoParietal | Frontal pole |
| 319 | R | 254.779 | 23.5 59.1 4.9 | FrontoParietal | Frontal pole |
| 320 | R | 96.1945 | 30.9 52.2 9.9 | FrontoParietal | Frontal pole |
| 327 | R | 384.2628 | 42.4 19.5 48.2 | FrontoParietal | Middle frontal gyrus |
| 328 | R | 142.111 | 38.9 9.6 42.7 | FrontoParietal | Middle frontal gyrus,  Precentral gyrus |

**Supplementary Table 15** Head motion estimation of mean Framewise displacement (FD, mm) in the dataset divided by the stratified tumor group.

|  | ***Head motion*** | | |  |  |  |  |  |
| --- | --- | --- | --- | --- | --- | --- | --- | --- |
|  | *left-hemisphere gliomas* | *right-hemisphere gliomas* | *low-grade gliomas* | *high-grade gliomas* | *Stats*  *[p-value, t-value]* |  |  |  |
| ***Patients*** | 15 | 13 | 11 | 17 |  |  |  |  |
| ***Mean FD (mm)*** | 0.22± 0.15 | 0.19± 0.13 | 0.18±0.13 | 0.22± 0.15 | **Hemisphere** [0.45, 0.75]  **Grade** [0.34, 0.95] |  |  |  |

**Figure S1**

Surface visualization of *Hubs* selection from Gordon et al., 2016 parcellation.

**
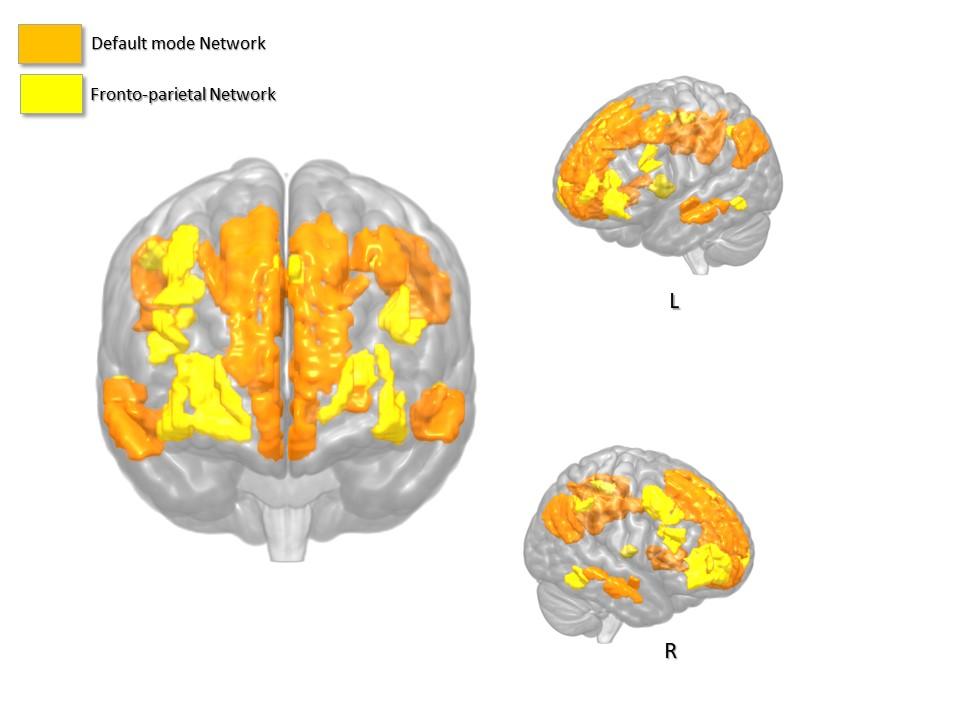
**

**Figure S2**

Probabilistic map of tumor localizations displaced on the surface and overlaid on the functional parcellation in high-grade gliomas (Panel A, HGG, N=17) low-grade gliomas (Panel B, LGG, N=11).


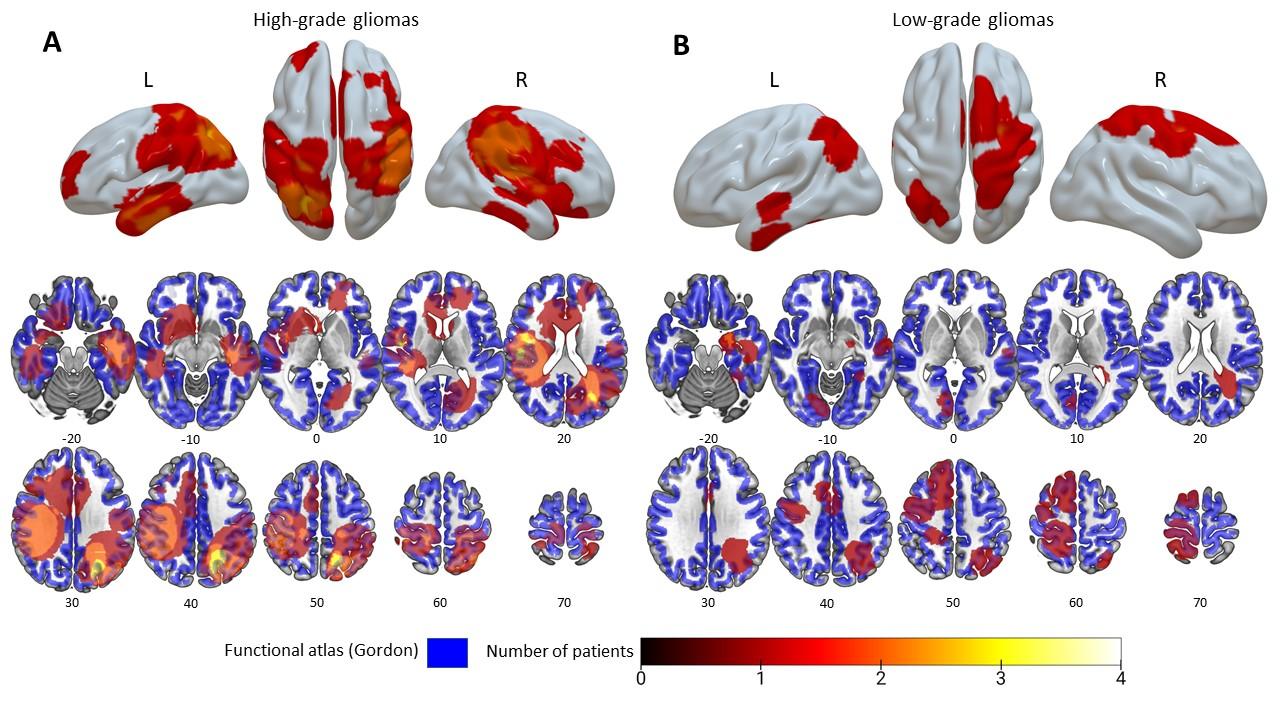


**Figure S3**

Prediction of the neuropsychological profile from longitudinal changes in functional connectivity metrics.

This figure depicts the interactions effect for the neuropsychological profile of brain tumor patients, regardless WHO tumor grade (low-grade gliomas (LGG) and tumor lateralization (e.g. left and right hemisphere) by plotting the quartiles of the distribution of each FC metric. In the top row, fixed interaction effect of *time* with default mode network (DMN) is shown (A) for Trial Making Tests (TMT-A and TMT-B). In the second row, fixed interaction effect of *time* with fronto-parietal network (FTPN) is shown (B) for Trial Making Test A.


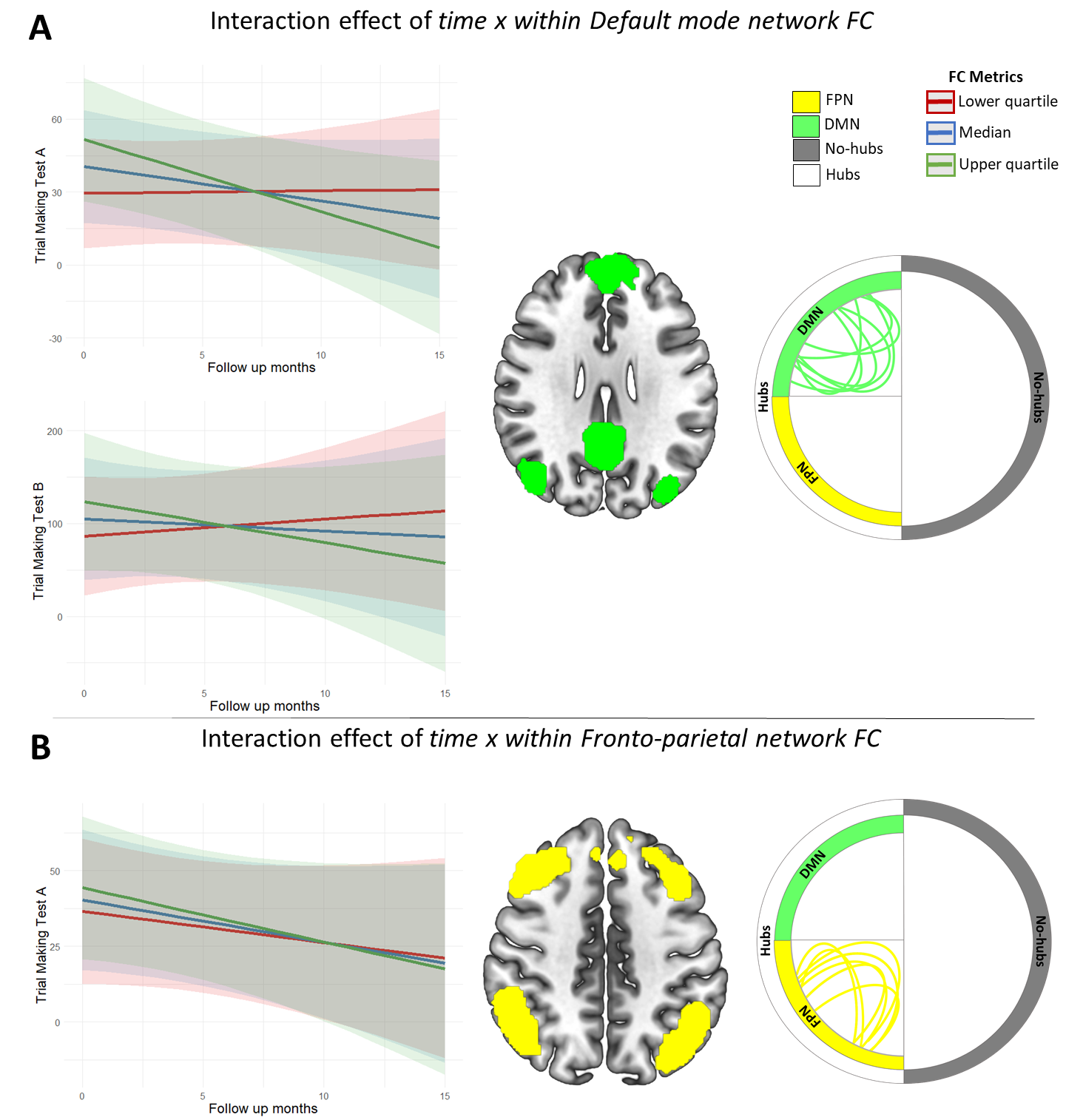


**References**

Briganti, C., Sestieri, C., Mattei, P. A., Esposito, R., Galzio, R. J., Tartaro, A., … Caulo, M. (2012). Reorganization of functional connectivity of the language network in patients with brain gliomas. *American Journal of Neuroradiology*, *33*(10), 1983–1990. https://doi.org/10.3174/ajnr.A3064

Caffarra, P., Vezzadini, G., Dieci, F., Zonato, F., & Venneri, A. (2002). Rey-Osterrieth complex figure: Normative values in an Italian population sample. *Neurological Sciences*, *22*(6), 443–447. https://doi.org/10.1007/s100720200003

Carlesimo, G. A., Caltagirone, C., Gainotti, G., Facida, L., Gallassi, R., Lorusso, S., … Parnett, L. (1996). The mental deterioration battery: Normative data, diagnostic reliability and qualitative analyses of cognitive impairment. *European Neurology*, *36*(6), 378–384. https://doi.org/10.1159/000117297

Correa, D. D., DeAngelis, L. M., Shi, W., Thaler, H. T., Lin, M., & Abrey, L. E. (2007). Cognitive functions in low-grade gliomas: Disease and treatment effects. *Journal of Neuro-Oncology*, *81*(2), 175–184. https://doi.org/10.1007/s11060-006-9212-3

Correa, D. D., Shi, W., Thaler, H. T., Cheung, A. M., DeAngelis, L. M., & Abrey, L. E. (2008). Longitudinal cognitive follow-up in low grade gliomas. *Journal of Neuro-Oncology*, *86*(3), 321–327. https://doi.org/10.1007/s11060-007-9474-4

De Baene, W., Rutten, G. J. M., & Sitskoorn, M. M. (2019). Cognitive functioning in glioma patients is related to functional connectivity measures of the non-tumoural hemisphere. *European Journal of Neuroscience*, *50*(12), 3921–3933. https://doi.org/10.1111/ejn.14535

Derks, J., Dirkson, A. R., de Witt Hamer, P. C., van Geest, Q., Hulst, H. E., Barkhof, F., … Douw, L. (2017). Connectomic profile and clinical phenotype in newly diagnosed glioma patients. *NeuroImage: Clinical*, *14*, 87–96. https://doi.org/10.1016/j.nicl.2017.01.007

Derks, J., Kulik, S., Wesseling, P., Numan, T., Hillebrand, A., van Dellen, E., … Douw, L. (2019). Understanding cognitive functioning in glioma patients: The relevance of IDH-mutation status and functional connectivity. *Brain and Behavior*, *9*(4), 1–9. https://doi.org/10.1002/brb3.1204

Esposito, R., Mattei, P. A., Briganti, C., Romani, G. L., Tartaro, A., & Caulo, M. (2012). Modifications of default-mode network connectivity in patients with cerebral glioma. *PLoS ONE*, *7*(7). https://doi.org/10.1371/journal.pone.0040231

Gainotti, G., Marra, C., & Villa, G. (2001). A double dissociation between accuracy and time of execution on attentional tasks in Alzheimer’s disease and multi-infarct dementia. *Brain*, *124*(4), 731–738. https://doi.org/10.1093/brain/124.4.731

Giovagnoli, A. R., Del Pesce, M., Mascheroni, S., Simoncelli, M., Laiacona, M., & Capitani, E. (1996). Trail Making Test: Normative values from287 normal adult controls. *Italian Journal of Neurological Sciences*, *17*(4), 305–309. https://doi.org/10.1007/BF01997792

Gordon, E. M., Laumann, T. O., Adeyemo, B., Huckins, J. F., Kelley, W. M., & Petersen, S. E. (2016). Generation and Evaluation of a Cortical Area Parcellation from Resting-State Correlations. *Cerebral Cortex*, *26*(1), 288–303. https://doi.org/10.1093/cercor/bhu239

Habets, E. J. J., Kloet, A., Walchenbach, R., Vecht, C. J., Klein, M., & Taphoorn, M. J. B. (2014). Tumour and surgery effects on cognitive functioning in high-grade glioma patients. *Acta Neurochirurgica*, *156*(8), 1451–1459. https://doi.org/10.1007/s00701-014-2115-8

Harris, R. J., Bookheimer, S. Y., Cloughesy, T. F., Kim, H. J., Pope, W. B., Lai, A., … Ellingson, B. M. (2014). Altered functional connectivity of the default mode network in diffuse gliomas measured with pseudo-resting state fMRI. *Journal of Neuro-Oncology*, *116*(2), 373–379. https://doi.org/10.1007/s11060-013-1304-2

Lahti, J., Saunamäki, T., Salo, J., Niemelä, A., & Jehkonen, M. (2018). Cognitive Impairment and Recovery in Meningiomas and Low-Grade Gliomas. *Journal of Behavioral and Brain Science*, *08*(08), 473–484. https://doi.org/10.4236/jbbs.2018.88029

Laiacona, M., Barbarotto, R., Trivelli, C., & Capitani, E. (1993). Dissociazioni semantiche intercategoriali: descrizione di una batteria standardizzata e dati normativi. [Category specific semantic defects: A standardised test with normative data.]. *Archivio Di Psicologia, Neurologia e Psichiatria*, Vol. 54, pp. 209–248. Italy: Universita Cattolica del Sacro Cuore.

Maesawa, S., Bagarinao, E., Fujii, M., Futamura, M., Motomura, K., Watanabe, H., … Wakabayashi, T. (2015). Evaluation of resting state networks in patients with gliomas: Connectivity changes in the unaffected side and its relation to cognitive function. *PLoS ONE*, *10*(2), 1–13. https://doi.org/10.1371/journal.pone.0118072

Messé, A. (2020). Parcellation influence on the connectivity-based structure–function relationship in the human brain. *Human Brain Mapping*, *41*(5), 1167–1180. https://doi.org/10.1002/hbm.24866

Novelli, G., Papagno, C., Capitani, E., Laiacona, M., & al, et. (1986). Tre test clinici di ricerca e produzione lessicale. Taratura su sogetti normali. [Three clinical tests to research and rate the lexical performance of normal subjects.]. *Archivio Di Psicologia, Neurologia e Psichiatria*, Vol. 47, pp. 477–506. Italy: Universita Cattolica del Sacro Cuore.

Orsini, Grossi, Capitani, Laiacona, Papagno, C., & Vallar, G. (1987). Verbal and spatial immediate memory span. *The Italian Journal of Neurological Sciences*, *8*, 539–548.

Robinson, G., Shallice, T., Bozzali, M., & Cipolotti, L. (2012). The differing roles of the frontal cortex in fluency tests. *Brain*, *135*(7), 2202–2214. https://doi.org/10.1093/brain/aws142

Satoer, D., Vincent, A., Smits, M., Dirven, C., & Visch-Brink, E. (2013). Spontaneous speech of patients with gliomas in eloquent areas before and early after surgery. *Acta Neurochirurgica*, *155*(4), 685–692. https://doi.org/10.1007/s00701-013-1638-8

SPINNLER, & H. (1987). Standardizzazione e taratura italiana di test neuropsicologici. *Ital J Neurol Sci*, *6*, 21–120.

Tuovinen, N., Pasquale, F. De, Caulo, M., Caravasso, C. F., Giudice, E., Miceli, R., … Sabatini, U. (2016). Transient effects of tumor location on the functional architecture at rest in glioblastoma patients : three longitudinal case studies. *Radiation Oncology*, 1–18. https://doi.org/10.1186/s13014-016-0683-x

Tzourio-Mazoyer, N., Landeau, B., Papathanassiou, D., Crivello, F., Etard, O., Delcroix, N., … Joliot, M. (2002). Automated anatomical labeling of activations in SPM using a macroscopic anatomical parcellation of the MNI MRI single-subject brain. *NeuroImage*, *15*(1), 273–289. https://doi.org/10.1006/nimg.2001.0978

van Dokkum, L. E. H., Moritz Gasser, S., Deverdun, J., Herbet, G., Mura, T., D’Agata, B., … le Bars, E. (2019). Resting state network plasticity related to picture naming in low-grade glioma patients before and after resection. *NeuroImage: Clinical*, *24*(September), 102010. https://doi.org/10.1016/j.nicl.2019.102010

van Loenen, I. S., Rijnen, S. J. M., Bruijn, J., Rutten, G. J. M., Gehring, K., & Sitskoorn, M. M. (2018). Group Changes in Cognitive Performance After Surgery Mask Changes in Individual Patients with Glioblastoma. *World Neurosurgery*, *117*, e172–e179. https://doi.org/10.1016/j.wneu.2018.05.232

Xu, H., Ding, S., Hu, X., Yang, K., Xiao, C., Zou, Y., … Qian, Z. (2013). Reduced efficiency of functional brain network underlying intellectual decline in patients with low-grade glioma. *Neuroscience Letters*, *543*, 27–31. https://doi.org/10.1016/j.neulet.2013.02.062

Zacà, D., Jovicich, J., Corsini, F., Rozzanigo, U., Chioffi, F., & Sarubbo, S. (2018). ReStNeuMap: a tool for automatic extraction of resting-state functional MRI networks in neurosurgical practice. *Journal of Neurosurgery*, 1–8. https://doi.org/10.3171/2018.4.jns18474
